# Clinical Factors on Dental Implant Fractures: A Systematic Review

**DOI:** 10.3390/dj12070200

**Published:** 2024-06-28

**Authors:** Mattia Manfredini, Pier Paolo Poli, Luca Giboli, Mario Beretta, Carlo Maiorana, Matteo Pellegrini

**Affiliations:** 1 Department of Biomedical, Surgical and Dental Sciences, University of Milan, 20122 Milan, Italy; mattia.manfredini@unimi.it (M.M.); luca.giboli@unimi.it (L.G.); mario.beretta@unimi.it (M.B.); carlo.maiorana@unimi.it (C.M.); matteo.pellegrini@unimi.it (M.P.); 2 Implant Center for Edentulism and Jawbone Atrophies, Maxillofacial Surgery and Dental Unit, Fondazione IRCCS Ca’ Granda Ospedale Maggiore Policlinico, 20122 Milan, Italy

**Keywords:** clinical factors, clinical studies, dental implant, dentistry, fractures, implantology

## Abstract

Dental implant fractures pose a significant challenge to long-term treatment success. This systematic review aims to comprehensively examine the clinical factors influencing dental implant fractures (IFs). Furthermore, strategies to choose the right type of implant and prevent this complication are addressed. A systematic search was conducted across PubMed, Scopus, and Web of Science databases. Eligible studies included retrospective case–control, prospective cohort studies, and clinical trials. The initial search yielded 361 articles, of which 312 were excluded being these reviews, case reports, irrelevant, or written in languages other than English. This left 49 articles, with only 6 meeting the eligibility criteria for an in-depth review. These studies, all retrospective case–control, examine implant characteristics, patient demographics, surgical and prosthetic variables, biomechanical and functional factors, clinical and procedural variables, complications and maintenance issues. The risk of bias was assessed as low using the ROBINS-I tool. Key findings suggest a correlation between implant diameter and structural resistance, with wider implants demonstrating reduced fracture risk. Additionally, posterior regions, especially molars and premolars, exhibit higher susceptibility to IFs due to increased masticatory forces. Implant design and material may considerably influence fracture risk, with conical implants and screw-retained prostheses showing higher vulnerability. Biomechanical overload, particularly in patients with bruxism, emerges as a primary contributing factor to IFs. Prosthesis type significantly influences fracture incidence, with cantilever prostheses posing a higher risk due to increased stress. Peri-implant bone loss is strongly associated with IFs, emphasizing the need for meticulous preoperative assessments and individualized management strategies. Future research should prioritize larger and heterogeneous populations with long-term follow-up and standardized methodologies to enhance the generalizability and comparability of findings. Randomized controlled trials and biomechanical studies under controlled conditions are also essential to elucidate the complex interactions contributing to IFs and developing effective prevention strategies. Additionally, integrating patient-reported outcomes may offer a comprehensive understanding of the impact of IFs on quality of life.

## 1. Introduction

Dental implants are a reliable solution for the replacement of single and multiple missing teeth, yielding favorable results in the long term [1].

The most common complication is peri-implant disease, a plaque-related inflammatory process limited initially to soft tissues, a state known as peri-implant mucositis. If not diagnosed early, it becomes irreversible following a non-linear accelerating pattern, involving the resorption of the supporting bone, a condition named peri-implantitis [2]. Hypersensitivity reactions to metals have also been associated with implant loss. These may arise in predisposed patients chronically exposed to metallic materials, including dental implants made of titanium alloys, and can determine bone loss and ultimately implant failure [3].

Apart from biological issues, another group of complications is related to the biomechanical aspect and includes the mechanical fractures. These can affect the prosthetic structure and substructure, the prosthetic screw, and, more rarely, the body of the implant itself.

Considering the latter, implant fracture (IF), occurring more frequently in the posterior sectors of the jaws, is one of the main biomechanical reasons for implant failure and consequent removal [4].

Although IFs are rare, typical signs should be known and recognized early. These include inflammatory reactions and bone loss, screw loosening, and eventually mobility of the implant-supported restoration [5]. IFs have been detected in long-term studies and linked to several causes, including biological, mechanical, and technical factors [6]. In this respect, biomechanical overload, design problems, and incorrect operative planning have been pointed out as potential factors leading to IF [7].

The mechanical patterns of IFs often involve a combination of tensile and compressive stresses that exceed the material’s fatigue limit. Studies have identified common fracture patterns, such as fractures occurring at the implant neck due to bending moments, and mid-body fractures resulting from axial loading and cyclic fatigue. Understanding these patterns is crucial in order to improve implant design and surgical protocols and mitigate such complication [7].

Lack of frequency studies and clinical reports on IF patterns indicate, however, that the evidence in this field is scarce.

A recent systemic review by Verma et al. [8] was performed to address the paucity of clinical reviews concerning the simultaneous evaluation of mechanical complications associated with implants and their effect on prosthesis survival. The authors concluded that the overall prevalence of mechanical failures may vary between 5.6% and 7.7%, comparable to biological and aesthetic complications. The most common complications are screw misalignment, followed by screw fracture for implant-supported prostheses. Finally, the maxillary arch resulted more susceptible to mechanical complications and failures compared to the mandible. Other authors have focused on the causes of screw fracture of implant abutments, suggesting different techniques for their retrieval [9]. Others assessed the fracture resistance of zirconia implants in the anterior region, evaluating whether they could be a viable alternative to titanium implants [10].

However, as previously mentioned, there seems to be a lack of evidence in the current literature concerning the clinical factors leading to IFs. In particular, there are no systematic reviews to the best of the authors’ knowledge addressing the incidence and causes of this complication. Thus, the present systematic review aimed to provide a comprehensive overview of the factors involved in IFs, offering useful insights for a more informed clinical practice and targeted preventive strategies.

## 2. Materials and Methods

### 2.1. Focused Question

What are the clinical and mechanical factors influencing osseointegrated implant fractures?

### 2.2. Eligibility Criteria

The inclusion criteria considered for this review were (I) study design—retrospective and prospective cohort studies, case–control studies, and clinical trials; (II) human participants of any age who have undergone dental implant procedures; (III) interventions—dental implant fractures or failures, examining factors such as occlusal overload, implant characteristics, prosthetic planning, biomechanical influences, and complications related to dental implants; (IV) outcome—occurrence, risk factors, patterns, or causes of dental implant fractures; (V) studies published in the English language; (VI) studies published between year 2000 and 2023. The analysis was limited to studies that satisfied all the inclusion criteria, while the exclusion criteria comprised the following aspects: (I) abstracts of articles published in non-English languages; (II) duplicate studies; (III) studies lacking detailed information on dental implant fractures, their causes, or associated factors or not corresponding to the abstract’s content; (IV) ex vivo or experimental animal studies; (V) studies without ethics committee approval; and (VI) narrative, systematic, or meta-analysis reviews.

### 2.3. Search Strategy

A three-stage search process was executed following the methodology described by the Joanna Briggs Institute (JBI) for systematic reviews. Initially, preliminary and restricted exploration was carried out using PubMed (MEDLINE), Scopus, and Web of Science (WoS). Subsequently, the relevant terminology was extracted from the articles to formulate an all-encompassing research strategy. Finally, the reference lists of all articles were searched to identify any additional pertinent research [11].

The PICO model (Table 1) (Population, Intervention, Comparison, Outcome) was used to conduct this review, through a literature search of the PubMed (MEDLINE) and Scopus electronic databases, based on the following three aspects: population (participants of any age who have undergone dental implant procedures), concept (dental implant fractures or failures), and context (without confinement to any specific cultural or environmental component). Scrutiny of study abstracts investigating dental implant fractures or failures, examining factors such as occlusal overload, implant characteristics, prosthetic planning, biomechanical influences, and complications related to dental implants was conducted. Throughout this comprehensive literature review, adherence was maintained to the preferred reporting items for systematic reviews (PRISMA) consensus, as depicted in Table S1 (Supplementary Materials) [12].

### 2.4. Research

Electronic exploration was performed using the PubMed (MEDLINE), Scopus, and Web of Science (WoS) databases using the following string: (“Dental Implants” [MeSH]) AND (“fracture*” [title] OR “Break*” [title] OR “Crack” [title] OR “damage” [title] OR “Shatter” [title] OR “Rupture” [title] OR “Fragmentation” [title]). Articles published between 2000 and 2023 were included. Data were extracted between November 2023 and January 2023, and a final search was conducted on 4 January 2024. Any duplicate entries in the databases were identified and subsequently eliminated using the EndNote Web reference manager software (version 20) by Clarivate Analytics, based in Philadelphia, PA, USA.

The search was conducted by two reviewers (L.G., and M.P.). Any disparities that emerged during the review were resolved by consensus. For complex cases, four additional reviewers (M.M., M.B., C.M., and P.P.P.) were consulted. The initial phase of screening involved the assessment of article titles and abstracts, excluding irrelevant studies. Subsequently, the relevant articles underwent a comprehensive evaluation involving thoroughly examining their full content. The outcomes were carefully recorded, and similar studies that met the predetermined inclusion criteria were identified and incorporated in this review.

The present protocol was registered on the Open Science Framework platform (Registration DOI https://doi.org/10.17605/OSF.IO/25MZ7, accessed on 27 March 2024).

### 2.5. Data Extraction

Data extraction included study characteristics, study design, number of participants, implant characteristics, clinical factors registered during the study, type of analysis conducted on the sample, outcomes measured, and key findings. Following the review of the publications, a spreadsheet was generated and subsequently updated sequentially. The data were extracted from the published articles; the authors of the included studies were not contacted for more information or raw data.

The collected data were organized into tables, which provided a structured presentation of the information: the name of the first author of the article and the year of publication, the type of implant used, the factors investigated, the follow-up period, analyses performed on the samples, and the results of this analysis. Analyses performed on the samples mean the explanation of how some variables, such as implant position, marginal bone loss, implantoplasty, or implant-abutment connection affected the results.

### 2.6. Quality Assessment of Included Studies

In this study, the potential for bias in clinical studies was appraised through a qualitative analysis using the National Heart, Lung, and Blood Institute (NHLBI) (Bethesda, Maryland, United States) Quality Assessment Tools. This approach enabled a comprehensive and methodical evaluation of the quality and potential biases within the included studies, aiming to establish the dependability and credibility of the results [13].

## 3. Results

**Figure 1:**
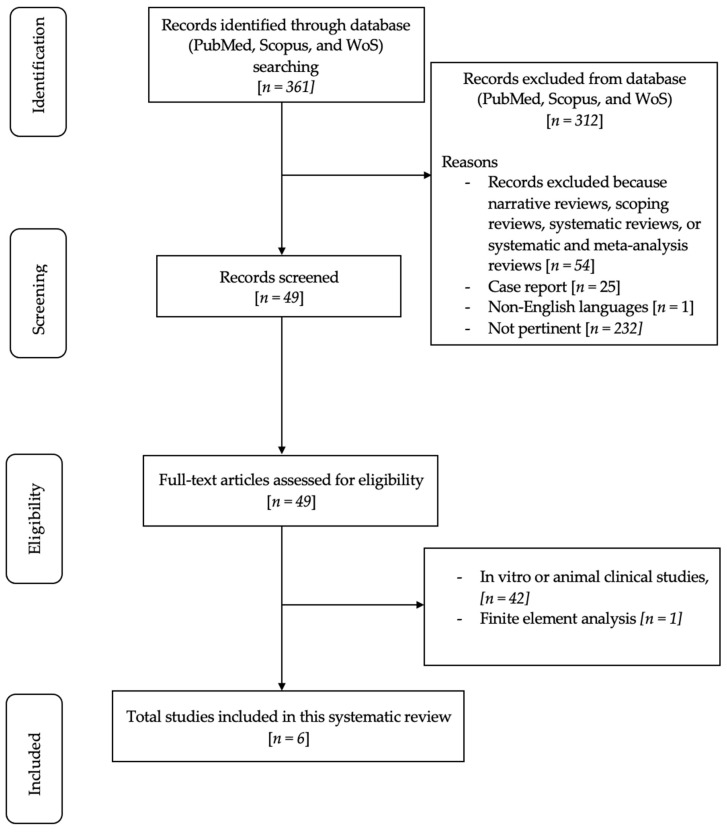
Flowchart of the review process.

The initial search using Medical Subject Headings (MeSH) terms resulted in 361 articles. A total of 312 articles were excluded for various reasons: 54 were narrative reviews, scoping reviews, systematic reviews, or systematic and meta-analyses, 25 were case reports, 1 article was published in the Hungarian language, and 232 were not relevant to the research topic. Following this initial screening, 49 articles were assessed based on their titles and abstracts. Among these, 49 full-text articles fulfilled the eligibility criteria and were included in the in-depth analysis. A total of 42 were excluded because they were related to in vitro or animal clinical studies, and 1 full-text article was excluded since the research topic concerned finite element analysis. Ultimately, six pertinent articles were comprehensively reviewed and scrutinized as part of this examination. Figure 1 shows a flowchart of the review procedure.

Table S2 (Supplementary Materials) displays the research papers not considered in this analysis and the explanations for their exclusion [14,15,16,17,18,19,20,21,22,23,24,25,26,27,28,29,30,31,32,33,34,35,36,37,38,39,40,41,42,43,44,45,46,47,48,49,50,51,52,53,54,55,56].

The studies included in this systematic review were all retrospective case–control studies [57,58,59,60,61,62].

NHLBI Quality Assessment Tool for Observational Cohort Studies is presented in Table S5 (Supplementary Materials).

The articles included considered the following aspects:

- Implant Characteristics: Diameter, Length, Design, Material, Cervical Feature, Microthread, Platform Switching, Connection Type;
- Patient Demographics: Age, Biological Sex;
- Surgical and Prosthetic Variables: Position in Jaw, Bone Characteristics, Timing;
- Prosthesis Characteristics: Type of Prosthesis, Type of Retention, Material of Prosthesis;
- Biomechanical and Functional Factors: Alignment, Jaw Relation, Functional Duration, Marginal Bone Loss, GBR, Screw Loosening, Screw Fracture, Fractured Implant Bodies;
- Clinical and Procedural Variables: Type of Abutment, Type of Implant-Abutment Connection, Healing Type, Opposing Tooth Type;
- Complications and Maintenance: Loosening and Fracture of Abutment Screws, Fractured Implant Bodies, Time of Onset of Complication.

### Risk of Bias

The assessment of bias risk in the articles included in this review was conducted using the Risk Of Bias in Non-Randomized Studies of Interventions (ROBINS-I) assessment tool (version 19 September 2016) [63]. Criteria for judging risk of bias in the ROBINS-I assessment tool [64] are outlined in Table S3 (Supplementary Materials). The outcomes of this assessment are shown in Table S4 (Supplementary Materials), revealing a low risk of bias.

Table 2 presents the baseline characteristics of patients, number of fixtures, and fractures included in this systematic review.

A detailed overview of the evidence obtained from the studies included in this review is presented in Table 3. This information includes the study design and aim, sample analysis, type of implant, predictive variables, and results drawn by authors of each study.

## 4. Discussion

In recent decades, dental implantology has advanced significantly in the replacement of missing teeth, greatly improving patients’ quality of life [63]. Despite the evident clinical success, IFs remain a critical complication that can severely compromise treatment outcomes [64].

Understanding the causes of IFs is pivotal to improve implant design, refine clinical practice, and maximize the longevity of implant-supported rehabilitations [65]. This systematic review explored the underlying mechanisms of IFs based on clinical studies, focusing on variables such as implant diameter and position, implant and prosthesis characteristics, type of mechanical load and implant-abutment connection, bone loss, regenerative procedures, and factors related to age, biological sex, and operator experience.

### 4.1. Implant Diameter and Structural Resistance Correlation

According to the current literature, a significant correlation exists between the diameter of the implant and its structural resistance. Tabrizi et al. [59] suggested that a reduced implant diameter, below 3.75 mm, could increase the risk of failure, making the implant more susceptible to IFs. However, it is worth noting that no significant correlation was found between implant diameter and fracture timing [59], as confirmed by Stoichkov et al. [61]. The lack of this correlation may suggest that factors other than the diameter itself could influence the structural resistance of implants.

A broader retrospective analysis conducted by Lee et al. [62] indicated that wide-diameter implants exhibit a significantly lower probability of fracture compared to narrow or regular diameters. This suggests that increasing implant diameter may contribute to greater resistance and stability, as indicated by higher Implant Stability Quotient (ISQ) values [62].

In another study, most fractures occurred in one-piece zirconia implants (Z-Look3) with a 3.25 mm diameter, while those with a 4 mm diameter were less susceptible to IFs [57]. This information provides valuable insights into the choice of diameter related to the specific material used to produce the implant [57].

Thus, implant diameter seems to play a role in structural resistance, with evidence supporting a direct relationship between increased diameter and enhanced stability, thereby reducing the risk of IFs.

### 4.2. Implant Position and Impact on IF Incidence

The incidence of IFs is significantly higher in molars and premolars compared to the anterior regions of the jaw. This trend is primarily attributed to the distribution of more intense masticatory forces and lateral movements with cusp inclination in the molar and premolar regions. Tabrizi’s study [59] supports this observation, emphasizing that the higher masticatory forces in the posterior area significantly contribute to the higher rates of IFs in these regions. Additionally, Cha et al. [58] reported a relatively higher prevalence of coronal fractures in the molar region, suggesting a specific vulnerability in this area despite the absence of horizontal IFs.

Moreover, Lee et al. [62] found that implants positioned in the anterior mandibular area had a lower risk of fracture compared to those positioned in other areas of the mandible. This finding indicates that, in addition to masticatory forces, the specific location of implants can influence their vulnerability.

Awareness of these trends is important in the planning and execution of implant procedures, guiding the choice of implant positions and potentially contributing to a reduction in the incidence of fractures. However, it is essential to consider other individual and clinical factors for a comprehensive risk assessment and personalized patient management.

### 4.3. Influence of Implant Design and Material on Fracture Risk

Based on the evidence available in the included studies, a complex picture emerges regarding implant-related factors. Tabrizi et al. [59] emphasized the criticality of the implant shape, highlighting an increased risk of fracture in conical implants and those with screw-retained prostheses. Particularly, screw-retained prostheses exhibit a higher incidence of technical complications and marginal bone loss. Stress distribution is a key element, with conical implants generating greater crestal stress compared to cylindrical implants of the same dimensions. Increasing the number of implants supporting the prosthesis is proposed as a strategy to reduce the risk of fractures.

Lee et al. [60] highlighted structural weakness as a relevant cause of fractures, particularly in specific designs of different implant systems. Horizontal fractures beyond the crestal module (Type III) were reported in implants with microthreads and macrothreads, indicating a higher risk of fractures due to structural weakness.

In 2019, Lee et al. [62] did not identify a significant correlation between implant material and fracture rates. However, in the case of zirconia implants, material improvements, as proposed by Kohal et al. [39], could contribute to greater resistance. These include zirconia tempered with alumina and modifications in implant geometry, as reported by Gahlert et al. [57].

Understanding the risk of IFs requires careful assessment of various factors, including implant shape, stress distribution, structural weakness, and the material used. The adoption of personalized approaches, considering these elements, could be fundamental in improving the resistance and longevity of dental implants.

### 4.4. Impact of Prosthesis Type on Dental Implant Fractures

The studies by Tabrizi et al. [59] and Stoichkov et al. [61] underline the significant impact of prosthesis type on the incidence of IFs. Tabrizi et al. [59] reported that cantilever prostheses are associated with a higher incidence of fractures due to the lever effect, generating significantly elevated stress on implants and predisposing them to fatigue and fracture. Moreover, cantilever prostheses were correlated with an earlier onset of fractures, suggesting an accelerated deterioration process.

Stoichkov et al. [61] confirmed the importance of prosthesis type in biomechanical complications, noting that most implant fractures occurred in patients with single crowns, while splinted crowns produced lower peri-implant tension. Implant fractures may be preceded by bone loss in the marginal area, especially when combined with cantilever extensions, highlighting the importance of carefully assessing prosthesis design and the potential presence of asymmetric loads.

An adequate design of the prosthesis contributes to prevent IFs. In this respect, specific attention to cantilever prostheses, avoiding or mitigating their biomechanical impact, could help reducing the risk of fatigue and thus IFs.

### 4.5. Biomechanical Overload as a Significant Factor in Dental Implant Fractures

Biomechanical overload, caused by various sources of occlusal stress and patient behaviors, is a significant factor associated with IFs. Cha et al. [58] highlighted the difference in the Cumulative Survival Rate (CSR) of Astra Tech implants between the molar region and the anterior and premolar regions, attributing this difference to higher masticatory forces present in the molar region. Flexural overload, generated by strong occlusal forces during chewing, was identified as the primary cause of implant coronal fractures.

Both Tabrizi et al. [59] and Lee et al. [60] emphasized that excessive occlusal load, particularly in molar and premolar sites, can be a potential cause of IFs. Lee et al. [60] specified that the cervical region of the implant was more prone to biomechanical stress, with horizontal and vertical fractures in the cervical portion representing most of the observed fractures.

Bruxism, as highlighted by Stoichkov et al. [61], has been identified as an etiological factor contributing to biological and biomechanical complications, representing 80% of IFs. Inadequate occlusion, producing unfavorable forces and stress concentrations on implants, is strongly associated with IFs. However, Lee et al. [62] indicated that no significant effect size was detected between screw loosening and implant fracture, suggesting the complexity of biomechanical interactions.

Gahlert et al. [57] emphasized that direct flexural loads from the palate to the vestibule or from the lingual to the buccal were identified as damaging causes in zirconia IFs. Marginal alignment of implants, when positioned more toward the vestibule, appears to increase susceptibility to IFs.

Managing occlusal load, controlling bruxism, and careful prosthesis design are key elements in preventing IFs. A detailed understanding of biomechanical interactions can guide clinical decisions to ensure the stability and durability of dental implants.

### 4.6. Crucial Role of Implant-Abutment Connection in Dental Implant Performance

The implant-abutment connection is an important aspect in evaluating the performance of dental implants. However, specific differences between various types of connections correlated to IFs did not significantly emerge in the examined full texts. Tabrizi et al. [59] did not report significant differences between different implant-abutment connections in relation to IFs. Nonetheless, the analysis of connections can provide relevant information about implant performance, suggesting that this aspect may influence the longevity and stability of dental implants.

Lee et al. [62] confirmed this trend, as no significant differences were found between two types of connections, namely internal hex and internal conical, in their relation to IFs. This reinforces the idea that, at least in terms of IFs, the choice between specific connections may not have a significant impact.

Tabrizi et al. [59] also noted that screw-retained prostheses presented more technical complications compared to those retained by cement, where greater marginal bone loss was also observed. This correlation could be attributed to stress transfer in implants, highlighting the importance not only of the type of connection but also of the fixation method in determining the long-term performance of dental implants.

Wang et al. [66] showed that increasing the taper angle significantly enhanced IF resistance, primarily due to the augmented thickness of the implant wall resulting from the enlargement of the taper angle. This effect was particularly pronounced when accommodating wide abutments with thin implant walls. However, for implants with small diameter abutments, the rate of increase in fracture resistance was relatively low or levels off with further increases in the taper angle. Furthermore, 3D-FEA stress analysis corroborated these findings, demonstrating variations in stress values among implants with different abutment taper angles and confirming the significance of taper angle in determining implant performance.

Although the implant-abutment connection is an important aspect to consider in planning and executing implant procedures, specific differences between types of connections may not be directly correlated to IFs. The choice of the connection type should therefore be weighed considering other factors such as the retention method and stress transfer to ensure the longevity and stability of dental implants over time.

### 4.7. Peri-Implant Bone Loss and Regeneration Procedures

The studies by Lee et al. [60,62] revealed a significant connection between peri-implant bone loss and fractures of dental implants, carrying important implications for the management and prognosis of such cases. The retrospective multicenter study by Lee et al. [60] on 19,087 implants pointed out that implant fractures were almost inevitably accompanied by vertical marginal bone loss, often associated with peri-implantitis. The severity of bone loss appeared to be directly correlated with the risk of fracture, with a rate approximately twice as high in cases with severe vertical bone loss exceeding 50% of the implant length. Furthermore, IFs occurred more frequently in implants placed in regenerated bone and two-stage implant surgery using bone grafts (Guided Bone Regeneration, GBR), especially when bone loss exceeded 50% of the implant length.

However, Lee et al. [62] provided an interesting perspective, indicating that implants with a history of bone graft and the presence of microthreads had a significantly lower risk of fracture than implants without a history of bone graft. This suggests that optimization of the hard tissue may play a protective role in implant stability over time, reducing the risk of IF.

Peri-implant bone loss emerges as a critical factor in IFs, with the degree of bone loss associated with the type of surgical intervention and the risk of complications. The importance of considering the history of bone graft underscores the significance of thorough preoperative assessments and personalized management strategies to maintain the stability and durability of dental implants over time.

### 4.8. Biological Sex, Surgical Expertise, and Other Parameters

The retrospective study by Cha et al. [58] suggested that, although biological sex did not show a statistically significant effect on CSR, most coronal fractures occurred in male patients. This implies a potentially higher risk for men compared to women, with fractures tending to occur earlier in male patients, presumably due to higher occlusal forces in this population. These data underscore the importance of considering biological sex as a possible risk factor in preoperative assessments.

Tabrizi et al. [59] indicated that other factors, such as the surgeon’s and prosthodontist’s experience, could play a significant role in IFs. The skills and expertise of the treatment team can be crucial in preventing complications.

Lee et al. [62] found that various parameters such as patient age, biological sex, implant length, cervical characteristics, connection type, and the presence of platform switching did not show significant correlations with fracture rates. This suggests that such factors might have a lesser influence on the risk of fracture.

Implantoplasty is a procedure that could lead to a decrease in the resistance of the implant structure [67]. While clinical studies on this topic are lacking, in vitro studies present conflicting results. Costa-Berenguer et al. [68] reported that implantoplasty did not significantly alter the fracture resistance of standard-diameter externally connected implants. Conversely, Camps-Font et al. [69] found that implantoplasty in small-diameter implants reduced implant wall thickness and fracture resistance, varying with the implant-abutment connection. Leitão-Almeida et al. [17] also suggested that implantoplasty significantly reduced the fracture resistance of implants with a crown-to-implant ratio of 2.5:1.

The findings from these studies emphasize the importance of considering various factors, including biological sex and the skills of the treatment team, in assessing the risk of IFs.

### 4.9. Limitations and Future Studies

The review has several limitations. The sample sizes and demographics in the studies differ, potentially impacting the generalizability of the findings. Larger and more diverse study populations would enhance the robustness of the conclusions. In addition, the follow-up in the studies varies, and longer-term observations are of paramount importance to understand the true incidence of IFs over time. Finally, the lack of standardization in reporting IFs and the heterogeneous methodologies used across the studies may hinder the ability to compare the results accurately.

Future biomechanical studies under controlled conditions could provide a clearer understanding of how occlusal forces contribute to implant fractures. This could involve simulated chewing forces and various prosthesis configurations. Furthermore, implementing randomized controlled trials (RCTs) with well-matched control groups could help establish causal relationships between various factors and implant fractures, minimizing confounding variables. Finally, future studies could include patient-reported outcomes, such as satisfaction and quality of life, to provide a more holistic understanding of the impact of implant fractures on individuals.

### 4.10. Result Summary Table

Based on the summary of evidence presented in Table 4, several factors influence implant fractures (IFs) in dental implants. 

A reduced implant diameter is associated with an increased risk of IF, although it does not affect fracture time in titanium implants. Conversely, wide-diameter zirconia implants show a lower probability of fracture. Implant position also plays a critical role, with molars and premolars experiencing higher IF incidence due to masticatory forces, while the anterior mandibular area exhibits a lower fracture risk. 

Implant design and material contribute significantly to IFs; conical implants and screw-retained prostheses have a higher fracture risk, and implants with microthreads may present structural weaknesses. 

Prosthesis type is another crucial factor, as cantilever prostheses and single crowns are more prone to fractures than splinted crowns. 

Biomechanical overload, characterized by high masticatory forces and bruxism, is a major cause of IFs, though no significant correlation is observed between screw loosening and IFs. 

The type of implant-abutment connection shows no significant differences in IF risk. Severe peri-implant bone loss increases fracture risk, whereas a history of bone grafting is linked to a lower risk. Biological sex influences IF incidence, with males showing higher rates due to stronger occlusal forces. 

Lastly, surgeon and prosthodontist expertise, implantoplasty, implant wall thickness, and implant-abutment connection all contribute to varying fracture resistance in implants.

## 5. Conclusions

This systematic review underscores the multifaceted nature of IFs and their significant impact on dental implantology. The correlation between implant diameter and structural resistance reveals that wider implants offer greater stability, reducing fracture risk. Additionally, the posterior regions, particularly molars and premolars, are more susceptible to IFs due to higher masticatory forces.

Implant design and material play major roles in fracture risk, with conical implants and screw-retained prostheses showing higher vulnerability. While implant material improvements such as tempered zirconia enhance resistance, no significant correlation between material type and fracture rates was found. Prosthesis type also significantly influences fracture incidence, with cantilever prostheses posing a higher risk due to increased stress.

Biomechanical overload, particularly in patients with bruxism and high occlusal forces, is a primary factor in IFs. Effective management of occlusal load and careful prosthesis design is essential for prevention. The implant-abutment connection’s impact on IFs appears less significant, although the retention method and stress transfer are important considerations.

Peri-implant bone loss is strongly linked to IFs, with severe bone loss and history of bone grafts influencing fracture rates. The review highlights the need for comprehensive preoperative assessments and personalized management strategies to enhance implant stability and longevity.

Future research should focus on larger, diverse populations, long-term follow-up, and standardized methodologies to improve the generalizability and comparability of findings. Randomized controlled trials and biomechanical studies under controlled conditions are necessary to elucidate the complex interactions contributing to IFs and to develop effective prevention strategies. Including patient-reported outcomes will provide a more comprehensive understanding of the impact of IFs on quality of life.

## Figures and Tables

**Table 1:** This table outlines the PICO model followed.

1. Participants/population: participants of any age who have undergone dental implant procedures
2. Intervention/exposure: dental implant fractures or failures
3. Comparison/control: dental implant without fractures or failures
4. Outcomes: clinical factors related to dental implant fractures

**Table 2:** Baseline characteristics of patients, number of fixtures, and fractures included in selected studies.

References (Authors, Year and Publication Country)	No. of Patients and Women (%)	Mean Age ± SD and/or Range (Years)	Inclusion and Exclusion Criteria	No. of Fixtures and Jaw Implant Position (%)	No. of Fractures and Jaw Implant Position (%)
Gahlert et al., 2012 Germany [57]	79; N.R.	N.R.	Inclusion criteria: N.R. Exclusion criteria: N.R.	170 N.R.	13 Maxilla, 11 (84.62%) Mandible, 2 (15.38%)
Cha et al., 2013 South Korea [58]	120; 63 (52.5%)	47 (18.8–81.1)	Inclusion criteria: N.R. Exclusion criteria: N.R.	136 Maxilla, 70 (51.5%) Mandible, 66 (48.5%)	11 Maxilla, 3 (27.27%) Mandible, 8 (72.73%)
Tabrizi et al., 2017 Iran [59]	6051; N.R.	N.R.	Inclusion criteria: missing teeth and dental implant treatment Exclusion criteria: dental implant fracture due to trauma	18,700 N.R.	37 Maxilla, 12 (32.43%) Mandible, 25 (67.57%)
Lee et al., 2018 South Korea [60]	8501; 663 (7.80%)	62.5 (27–81)	Inclusion criteria: N.R. Exclusion criteria: failure of initial and early osseointegration	19,087 Maxilla, 8528 (44.7%) Mandible, 10,559 (56.3%)	92 (only 70 analyzed) Maxilla, 32 (45.7%) Mandible, 38 (54.3%)
Stoichkov et al., 2018 Bulgaria [61]	101; 46 (45.5%)	N.R.	Inclusion criteria: N.R. Exclusion criteria: N.R.	218 Maxilla, 94 (43.12%) Mandible, 124 (56.88%)	5 Maxilla, 2 (40.00%) Mandible, 3 (60.00%)
Lee et al., 2019 South Korea [62]	5124; 554 (10.81%)	63.15 ± 9.10	Inclusion criteria: N.R. Exclusion criteria: inaccurate chart recordings, external or one-piece connection systems, supported removable prostheses, failure of initial or early osseointegration	19,006 Maxilla, 9722 (51.15%) Mandible, 9284 (48.85%)	174 Maxilla, 88 (50.57%) Mandible, 86 (49.43%)

Abbreviations: N.R., not reported.

**Table 3:** Evidence of studies included in this review.

Authors and Year of Publication	Study Design and Aim	Sample Analysis	Type of Implant	Predictive Variables	Results
Gahlert et al., 2012 [57]	A 36.75 ± 5.34-month retrospective cohort study with no control group aimed to evaluate the failure mechanism of 13 fractured dental zirconia implants by clinical, macroscopic, and scanning electron microscopic (SEM) methods.	Macroscopic, Light microscopy, SEM analysis.	Z-Look3 one-piece zirconia implants 3.25, 4, and 5 mm.	Diameter, implant position, implant alignment, jaw relation, time to failure.	Fracture incidence was approximately 10%; 12 of the 13 fractured implants had a diameter of 3.25 mm. All fractures occurred due to mechanical overloading (bending loads). Direction of crack propagation was always from the palatal position, lingual towards buccal. Macroscopic and light microscopic examinations offered no evidence that fracture of the implants occurred due to machining marks that might have been created for the connection of crowns or frameworks after implantation. SEM examinations confirmed that all fractures occurred due to singular bending overload (so-called forced rupture).
Cha et al., 2013 [58]	A 5-year retrospective cohort study with no control group aimed to evaluate the long-term cumulative survival rate (CSR) and complication rates of a 4.0 mm-diameter internal connection implant (Microthread^TM^ Osseospeed^TM^) installed for single-tooth restoration.	Survival rates, complications.	MicroThread Osseospeed Astra Tech, 4.0-mm-diameter internal connection implant.	Implant position, biological sex, fixture length, timing of implant placement, type of healing, type of opposing tooth, type of abutment type of prosthesis retention material of prosthesis, most distal position.	The occurrence of complications was significantly related to implant position. The complication survival rates of the whole arch and the molar region were 91.9% and 87.6%, respectively, after 5 years of loading. Hence, the failure rate of the molar region was 12.4%. The CSR of the molar region was significantly lesser than that of the anterior and premolar regions (*p* = 0.037).
Tabrizi et al., 2017 [59]	A 14-year retrospective cohort study with no control group aimed to estimate the incidence of implant fractures and identify factors associated with fracture.	Survival rates, complications.	Not specified.	Demographic characteristics (age, biological sex), anatomy (location of implants), physical characteristic of implants (cylindrical or tapered, size, and shape), type of implant-abutment connection, type of prosthesis (single crown or multiple implants with fixed prosthesis or cantilever prosthesis), and type of retention (screw-retained or cement-retained prosthesis).	Tapered implants and screw-retained implants increased the HR in implant fracture. The incidence of implant fracture was higher in the molars and premolars than in the anterior of the jaws. Implant fracture occurred sooner in cantilevers, screw-retained crowns, and tapered fixtures. No difference was found among the four implant-abutment connections. A cantilever prosthesis significantly increases stress in the implant prosthesis, and this may lead to implant fracture due to fatigue.
Lee et al., 2018 [60]	A 9-year retrospective cohort study with no control group aimed to investigate the incidence of implant fractures (IF) and long-term follow-up of patients with multicenter collaboration to classify patterns of fracture and to evaluate related clinical factors.	Survival rates, complications.	Astra Tech [Osseo-Speed, Mannheim, Germany], BioHorizons [Maestro, Birmingham, USA], Bio-TIS [Spider, Seoul, Korea], Camlog Biotechnologies [Root-Line, Wimsheim, Germany], Damool Science [Damool, Daegu, Korea], Dentis [OneQ/Cleanant, Daegu, Korea], Osstem [GS/TS, Seoul, Korea], Nobel Biocare [Replace, Göteborg, Sweden], and Zimmer [TSV, Carls- bad, USA]. Materials that were 3.0–6.0 mm in diameter (narrow [<3.75 mm], standard [3.75–5.0 mm], wide [>5.0 mm]) and 9.0–13.0 mm in length (standard [9.0–13.0] and long [>13 mm]) were used for implant surgery.	Patient variables (sex and age) and implant variables (implant system, diameter, length, position in the jaw, placement location, prosthesis type, marginal bone loss, functional duration, guided bone regeneration [GBR], screw loosening, and screw fracture).	Fractures were observed in a total of nine different implant systems, with an incidence of 0.4%. Most stress caused by biomechanical overloading is focused on the cervical area. No difference in diameters (small diameters were only placed in anterior zones) was found between mandible and maxilla. Peri-implantitis-induced marginal and vertical bone loss and structural weakness of the specific designs were major factors in IF.
Stoichkov et al., 2018 [61]	A 3–10-year retrospective cohort study with no control group aimed to analyze the possible causative factors contributing to implant body fracture.	Survival rates, complications.	TBR Connect, Periosave-M, and Z1-M (TBR Implants Group), Ankylos (Dentsply Implants), and Straumann Bone Level Implants (Institut Straumann) 3.2 to 6 mm.	Available bone volume, location of the dental implants and their inclinations (mesiodistal and buccolingual), type of prosthetic restorations (i.e., single crowns, splinted crowns, implant-supported fixed dental prostheses [FDPs], and tooth-implant-supported FDPs]), presence of cantilever extensions, type of connection between the implant and the abutment, size of the used diameters, degree of crestal resorption, loosening and fracture of the abutment screws, fractured implant bodies, as well as the time of onset of the complication.	Occlusal overload due to bruxism or to inappropriate or inadequate occlusion as single factors or a combination of these factors during the first years after the functional load can cause implant fracture. Fracture of the implant body more frequently occurred with single crowns than with other implant-supported FDPs.
Lee et al., 2019 [62]	A 12-year retrospective cohort study with no control group aimed to determining the fracture rate and risk indicator of internal connection implants installed in a single center.	Survival rates, complications.	Internal connection implants.	Age and sex of the patient; length of the fixture, diameter of the fixture, location of the implant, bone graft (presence or absence); fixture material (commercially pure titanium Grade 4 or titanium–aluminum–vanadium (Ti-6Al-4V)); cervical feature (polished or unpolished); type of implant connection (butt joint or conical joint); microthread (presence or absence); and platform switching (presence or absence). Dates for fixture installation, prosthesis delivery, and final visit.	Wide-diameter implants had a lower risk of fracture than narrow or regular-diameter implants; the absence of microthreads or bone grafts led to a higher risk of fracture than the presence of these features; the mandibular anterior area had a lower risk of fracture than other sites.

Abbreviations: CSR, long-term cumulative survival rate; FDPs, fixed dental prostheses; IF, implant fractures; SEM, scanning electron microscopic.

**Table 4:** Summary of evidence.

Influencing Factor	Key Findings
Implant Diameter	- Reduced diameter seems to increase IF risk but not fracture time in titanium implants. - Wide-diameter zirconia implants show lower fracture probability reduced diameter.
Implant Position	- Higher IF incidence in molars and premolars area due to masticatory forces. - Lower fracture risk in anterior mandibular area.
Implant Design and Material	- Conical implants and screw-retained prostheses have higher fracture risk. - Implants with microthreads may have structural weakness.
Prosthesis Type	- Cantilever prostheses are linked to higher fracture incidence. - Single crowns are more prone to fractures than splinted crowns.
Biomechanical Overload	- High masticatory forces and bruxism are significant IF causes. - No significant correlation shown between screw loosening and IF.
Implant-Abutment Connection	- No significant differences between connection types in IFs.
Peri-implant Bone Loss	- Severe bone loss increases fracture risk. - Bone graft history linked to lower fracture risk.
Biological Sex and Expertise	- Higher IF incidence in male patients presumably due to stronger occlusal forces. - Surgeon and prosthodontist expertise may play a role in preventing IFs.
Other Parameters	- Implantoplasty may reduce fracture resistance in small-diameter implants. - Implant wall thickness and fracture resistance vary with implant-abutment connection.

## Data Availability

Upon request to the corresponding author, the data are available for use. The protocol of the review was registered with the Open Science Framework (OSF) at https://doi.org/10.17605/OSF.IO/25MZ7 and registered from osf.io/3vsuj (accessed on 27 March 2024).

## Supplementary Materials

The following supporting information can be downloaded at: https://www.mdpi.com/article/10.3390/dj12070200/s1, Table S1: PRISMA 2020 Checklist; Table S2: Summary table of studies excluded in this systematic review; Table S3: Evidence of studies included in this systematic review; Table S3: Criteria for judging risk of bias in the ROBINS-I assessment tool; Table S4: Risk of bias of the studies included in this review through the ROBINS-I assessment tool; Table S5: The NHLBI Quality Assessment Tool for Observational Cohort Studies. [14,15,16,17,18,19,20,21,22,23,24,25,26,27,28,29,30,31,32,33,34,35,36,37,38,39,40,41,42,43,44,45,46,47,48,49,50,51,52,53,54,55,56,57,58,59,60,61,62,70].

## Abbreviations

CSR	Cumulative Survival Rate
FDPs	Fixed Dental Prostheses
FEA	Finite Element Analysis
GBR	Guided Bone Regeneration
IF	Implant Fracture
ISQ	Implant Stability Quotient (ISQ)
MeSH	Medical Subject Headings
NHLBI	National Heart, Lung, and Blood Institute
PCC	Population–Concept–Context
RCTs	Randomized Controlled Trials
ROBINS-I	Risk Of Bias in Non-Randomized Studies of Interventions
SEM	Scanning Electron Microscopic
WoS	Web of Science
