# Supplementary material for: Clinical Factors on Dental Implant Fractures: A Systematic Review
Source: Dent J (Basel). 2024 Jun 28;12(7):200. doi: 10.3390/dj12070200 (PMC11276356; doi:10.3390/dj12070200)
Supplement: Supplementary file 1 [file dentistry-12-00200-s001.zip › dentistry-2957732-supplementary.pdf]

Table S1. PRISMA 2020 Checklist

Section and Topic	Item #	Checklist item	Location where item is reported
**TITLE**			
Title	1	Identify the report as a systematic review.	Title
**ABSTRACT**			
Abstract	2	See the PRISMA 2020 for Abstracts checklist.	
**INTRODUCTION**			
Rationale	3	Describe the rationale for the review in the context of existing knowledge.	Initial introduction
Objectives	4	Provide an explicit statement of the objective(s) or question(s) the review addresses.	End of introduction
**METHODS**			
Eligibility criteria	5	Specify the inclusion and exclusion criteria for the review and how studies were grouped for the syntheses.	Dedicated section in M&M
Information sources	6	Specify all databases, registers, websites, organisations, reference lists and other sources searched or consulted to identify studies. Specify the date when each source was last searched or consulted.	Dedicated section in M&M
Search strategy	7	Present the full search strategies for all databases, registers and websites, including any filters and limits used.	Dedicated section in M&M
Selection process	8	Specify the methods used to decide whether a study met the inclusion criteria of the review, including how many reviewers screened each record and each report retrieved, whether they worked independently, and if applicable, details of automation tools used in the process.	Dedicated section in M&M
Data collection process	9	Specify the methods used to collect data from reports, including how many reviewers collected data from each report, whether they worked independently, any processes for obtaining or confirming data from study investigators, and if applicable, details of automation tools used in the process.	Dedicated section in M&M
Data items	10a	List and define all outcomes for which data were sought. Specify whether all results that were compatible with each outcome domain in each study were sought (e.g. for all measures, time points, analyses), and if not, the methods used to decide which results to collect.	Dedicated section in M&M
	10b	List and define all other variables for which data were sought (e.g. participant and intervention characteristics, funding sources). Describe any assumptions made about any missing or unclear information.	Dedicated section in M&M
Study risk of bias assessment	11	Specify the methods used to assess risk of bias in the included studies, including details of the tool(s) used, how many reviewers assessed each study and whether they worked independently, and if applicable, details of automation tools used in the process.	2 reviewers assessed the risk of bias – specified in M&M
Effect measures	12	Specify for each outcome the effect measure(s) (e.g. risk ratio, mean difference) used in the synthesis or presentation of results.	Mean difference (M&M)
Synthesis methods	13a	Describe the processes used to decide which studies were eligible for each synthesis (e.g. tabulating the study intervention characteristics and comparing against the planned groups for each synthesis (item #5)).	Type of intervention
	13b	Describe any methods required to prepare the data for presentation or synthesis, such as handling of missing summary statistics, or data	Procedure described

Section and Topic	Item #	Checklist item	Location where item is reported
		conversions.	M&M
	13c	Describe any methods used to tabulate or visually display results of individual studies and syntheses.	Procedure described M&M
	13d	Describe any methods used to synthesize results and provide a rationale for the choice(s). If meta-analysis was performed, describe the model(s), method(s) to identify the presence and extent of statistical heterogeneity, and software package(s) used.	Answer to PICO
	13e	Describe any methods used to explore possible causes of heterogeneity among study results (e.g. subgroup analysis, meta-regression).	N/A
	13f	Describe any sensitivity analyses conducted to assess robustness of the synthesized results.	N/A
Reporting bias assessment	14	Describe any methods used to assess risk of bias due to missing results in a synthesis (arising from reporting biases).	N/A
Certainty assessment	15	Describe any methods used to assess certainty (or confidence) in the body of evidence for an outcome.	Reported conclusions of the included studies
**RESULTS**			
Study selection	16a	Describe the results of the search and selection process, from the number of records identified in the search to the number of studies included in the review, ideally using a flow diagram.	Dedicated table
	16b	Cite studies that might appear to meet the inclusion criteria, but which were excluded, and explain why they were excluded.	Dedicated table
Study characteristics	17	Cite each included study and present its characteristics.	Dedicated table
Risk of bias in studies	18	Present assessments of risk of bias for each included study.	Dedicated table
Results of individual studies	19	For all outcomes, present, for each study: (a) summary statistics for each group (where appropriate) and (b) an effect estimate and its precision (e.g. confidence/credible interval), ideally using structured tables or plots.	Dedicated table
Results of syntheses	20a	For each synthesis, briefly summarise the characteristics and risk of bias among contributing studies.	Dedicated table
	20b	Present results of all statistical syntheses conducted. If meta-analysis was done, present for each the summary estimate and its precision (e.g. confidence/credible interval) and measures of statistical heterogeneity. If comparing groups, describe the direction of the effect.	N/A
	20c	Present results of all investigations of possible causes of heterogeneity among study results.	Dedicated table
	20d	Present results of all sensitivity analyses conducted to assess the robustness of the synthesized results.	Dedicated table
Reporting biases	21	Present assessments of risk of bias due to missing results (arising from reporting biases) for each synthesis assessed.	Dedicated table
Certainty of evidence	22	Present assessments of certainty (or confidence) in the body of evidence for each outcome assessed.	Dedicated table
**DISCUSSION**			
Discussion	23a	Provide a general interpretation of the results in the context of other evidence.	Followed

Section and Topic	Item #	Checklist item	Location where item is reported
	23b	Discuss any limitations of the evidence included in the review.	Followed
	23c	Discuss any limitations of the review processes used.	Followed
	23d	Discuss implications of the results for practice, policy, and future research.	Followed
**OTHER INFORMATION**			
Registration and protocol	24a	Provide registration information for the review, including register name and registration number, or state that the review was not registered.	DOI No 10.17605/OSF.IO/25MZ7
	24b	Indicate where the review protocol can be accessed, or state that a protocol was not prepared.	OSF
	24c	Describe and explain any amendments to information provided at registration or in the protocol.	N/A
Support	25	Describe sources of financial or non-financial support for the review, and the role of the funders or sponsors in the review.	None
Competing interests	26	Declare any competing interests of review authors.	None
Availability of data, code and other materials	27	Report which of the following are publicly available and where they can be found: template data collection forms; data extracted from included studies; data used for all analyses; analytic code; any other materials used in the review.	N/A

Table S2. Summary table of studies excluded in this scoping review**.**

Excluded Studies	Exclusion Reasons
Bufalá Pérez M. et al, 2022 [14]	In vitro study
Gehrke S.A. et al., 2022 [15]	In vitro study
Khorshidparast S. et al., 2023 [16]	In vitro study
Leitão-Almeida B. et al., 2020 [17]	In vitro study
Jorio I.C. et al., 2021 [18]	In vitro study
Aramburú J.S. et al., 2021 [19]	In vitro study
Bordin D. et al., 2017 [20]	In vitro study
Kim Y.J. et al., 2022 [21]	In vitro study
Streckbein P. et al., 2019 [22]	In vitro study
Burkhardt F. et al., 2021 [23]	In vitro study
Gehrke S.A., 2019 [24]	In vitro study
Agustín-Panadero R. et al., 2020 [25]	In vitro study
Leitão-Almeida B. et al., 2021 [26]	In vitro study
Bauer R. et al., 2021 [27]	In vitro study
Foong, J.K. et al., 2013 [28]	In vitro study

El-Mahdy M. et al., 2023 [29]	In vitro study
Kono K. et al., 2014 [30]	In vitro study
Asl H.G., et al., 2020 [31]	In vitro study
Emam M. et al., 2023 [32]	In vitro study
Igarashi K. et al., 2019 [33]	In vitro study
Freitas D.Q. et al., 2019 [34]	In vitro study
Zhai Z. et al., 2023 [35]	In vitro study
Vult von Steyern P. et al., 2005 [36]	In vitro study
Gehrke S.A. et al., 2014 [37]	In vitro study
Chong K.K. et al., 2014 [38]	In vitro study
Kohal R.J. et al., 2011 [39]	In vitro study
Bein L. et al., 2023 [40]	In vitro study
Lin J. et al., 2023 [41]	In vitro study
Rues S. et al., 2020 [42]	In vitro study
Katsavochristou A. et al., 2020 [43]	In vitro study
Vahnström M. et al., 2022 [44]	In vitro study
Sailer I. et al., 2009 [45]	In vitro study

Schmitter M. et al., 2010 [46]	In vitro study
Ghodsi S. et al., 2021 [47]	In vitro study
Gehrke S.A. et al., 2015 [48]	In vitro study
Giner S. et al., 2021 [49]	In vitro study
Wilmes B. et al., 2011 [50]	In vitro study
Att W. et al., 2006 [51]	In vitro study
Moorthy A. et al., 2022 [52]	In vitro study
Coppedê A.R. et al., 2009 [53]	In vitro study
Patankar A. et al., 2016 [54]	In vitro study
Bhagat J.A. et al., 2019 [55]	In vitro study
Takeshita K. et al., 2016 [56]	Finite element analysis

Table S3. Criteria for judging risk of bias in ROBINS-I assessment tool [70].

**1. Reaching risk of bias judgements for bias due to confounding**	
Low risk of bias (the study is comparable to a well-performed randomized trial with regard to this domain)	No confounding expected.
Moderate risk of bias (the study is sound for a non-randomized study with regard to this domain but cannot be considered comparable to a well-performed randomized trial)	(i) Confounding expected, all known important confounding domains appropriately measured and controlled for; and (ii) Reliability and validity of measurement of important domains were sufficient, such that we do not expect serious residual confounding.
Serious risk of bias (the study has some important problems)	(i) At least one known important domain was not appropriately measured, or not controlled for; or (ii) Reliability or validity of measurement of an important domain was low enough that we expect serious residual confounding.
Critical risk of bias (the study is too problematic to provide any useful evidence on the effects of intervention)	(i) Confounding inherently not controllable or (ii) The use of negative controls strongly suggests unmeasured confounding.
No information on which to base a judgement about risk of bias for this domain	No information on whether confounding might be present.
**2. Reaching risk of bias judgements for bias in selection of participants into the study**	
Low risk of bias (the study is comparable to a well-performed randomized trial with regard to this domain)	(i) All participants who would have been eligible for the target trial were included in the study; and (ii) For each participant, start of follow up and start of intervention coincided.
Moderate risk of bias (the study is sound for a non-randomized study with regard to this domain but cannot be considered comparable to a well-performed randomized trial)	(i) Selection into the study may have been related to intervention and outcome; and the authors used appropriate methods to adjust for the selection bias; or (ii) Start of follow-up and start of intervention do not coincide for all participants; and (a) the proportion of participants for which this was the case was too low to induce important bias; or (b) the authors used appropriate

	methods to adjust for the selection bias; or (c) the review authors are confident that the rate (hazard) ratio for the effect of intervention remains constant over time.
Serious risk of bias (the study has some important problems)	(i) Selection into the study was related (but not very strongly) to intervention and outcome; and This could not be adjusted for in analyses; or (ii) Start of follow up and start of intervention do not coincide; and A potentially important amount of follow-up time is missing from analyses; and The rate ratio is not constant over time.
Critical risk of bias (the study is too problematic to provide any useful evidence on the effects of intervention)	(i) Selection into the study was very strongly related to intervention and outcome; and This could not be adjusted for in analyses; or (ii) A substantial amount of follow-up time is likely to be missing from analyses; and The rate ratio is not constant over time.
No information on which to base a judgement about risk of bias for this domain	No information is reported about selection of participants into the study or whether start of follow up and start of intervention coincide.
**3. Reaching risk of bias judgements for bias in classification of interventions**	
Low risk of bias (the study is comparable to a well-performed randomized trial with regard to this domain)	(i) Intervention status is well defined; and (ii) Intervention definition is based solely on information collected at the time of intervention.
Moderate risk of bias (the study is sound for a non-randomized study with regard to this domain but cannot be considered comparable to a well-performed randomized trial)	(i) Intervention status is well defined; and (ii) Some aspects of the assignments of intervention status were determined retrospectively.
Serious risk of bias (the study has some important problems)	(i) Intervention status is not well defined; or (ii) Major aspects of the assignments of intervention status were determined in a way that could have been affected by knowledge of the outcome.

Critical risk of bias (the study is too problematic to provide any useful evidence on the effects of intervention)	(Unusual) An extremely high amount of misclassification of intervention status, e.g. because of unusually strong recall biases.
No information on which to base a judgement about risk of bias for this domain	No definition of intervention or no explanation of the source of information about intervention status is reported.
**4. Reaching risk of bias judgements for bias due to deviations from intended interventions**	
Low risk of bias (the study is comparable to a well-performed randomized trial with regard to this domain)	*Effect of assignment to intervention:* (i) Any deviations from intended intervention reflected usual practice; or (ii) Any deviations from usual practice were unlikely to impact on the outcome.<br>*Effect of starting and adhering to intervention:* The important co-interventions were balanced across intervention groups, and there were no deviations from the intended interventions (in terms of implementation or adherence) that were likely to impact on the outcome.
Moderate risk of bias (the study is sound for a non-randomized study with regard to this domain but cannot be considered comparable to a well-performed randomized trial)	*Effect of assignment to intervention:* There were deviations from usual practice, but their impact on the outcome is expected to be slight.<br>*Effect of starting and adhering to intervention:* (i) There were deviations from intended intervention, but their impact on the outcome is expected to be slight or (ii) The important co-interventions were not balanced across intervention groups, or there were deviations from the intended interventions (in terms of implementation and/or adherence) that were likely to impact on the outcome; and The analysis was appropriate to estimate the effect of starting and adhering to intervention, allowing for deviations (in terms of implementation, adherence and co-intervention) that were likely to impact on the outcome.

Serious risk of bias (the study has some important problems)	*Effect of assignment to intervention:* There were deviations from usual practice that were unbalanced between the intervention groups and likely to have affected the outcome.<br>*Effect of starting and adhering to intervention:* (i) The important co-interventions were not balanced across intervention groups, or there were deviations from the intended interventions (in terms of implementation and/or adherence) that were likely to impact on the outcome; and (ii) The analysis was not appropriate to estimate the effect of starting and adhering to intervention, allowing for deviations (in terms of implementation, adherence and co-intervention) that were likely to impact on the outcome.
Critical risk of bias (the study is too problematic to provide any useful evidence on the effects of intervention)	*Effect of assignment to intervention:* There were substantial deviations from usual practice that were unbalanced between the intervention groups and likely to have affected the outcome.<br>*Effect of starting and adhering to intervention:* (i) There were substantial imbalances in important co-interventions across intervention groups, or there were substantial deviations from the intended interventions (in terms of implementation and/or adherence) that were likely to impact on the outcome; and (ii) The analysis was not appropriate to estimate the effect of starting and adhering to intervention, allowing for deviations (in terms of implementation, adherence and co-intervention) that were likely to impact on the outcome.
No information on which to base a judgement about risk of bias for this domain	No information is reported on whether there is deviation from the intended intervention.
**5. Reaching risk of bias judgements for bias due to missing data**	

Low risk of bias (the study is comparable to a well-performed randomized trial with regard to this domain)	(i) Data were reasonably complete; or (ii) Proportions of and reasons for missing participants were similar across intervention groups; or (iii) The analysis addressed missing data and is likely to have removed any risk of bias.
Moderate risk of bias (the study is sound for a non-randomized study with regard to this domain but cannot be considered comparable to a well-performed randomized trial)	(i) Proportions of and reasons for missing participants differ slightly across intervention groups; and (ii) The analysis is unlikely to have removed the risk of bias arising from the missing data.
Serious risk of bias (the study has some important problems)	(i) Proportions of missing participants differ substantially across interventions; or Reasons for missingness differ substantially across interventions; and (ii) The analysis is unlikely to have removed the risk of bias arising from the missing data; or Missing data were addressed inappropriately in the analysis; or The nature of the missing data means that the risk of bias cannot be removed through appropriate analysis.
Critical risk of bias (the study is too problematic to provide any useful evidence on the effects of intervention)	(i) (Unusual) There were critical differences between interventions in participants with missing data; and (ii) Missing data were not, or could not, be addressed through appropriate analysis.
No information on which to base a judgement about risk of bias for this domain	No information is reported about missing data or the potential for data to be missing.
**6. Reaching risk of bias judgements for bias in measurement of outcomes**	
Low risk of bias (the study is comparable to a well-performed randomized trial with regard to this domain)	(i) The methods of outcome assessment were comparable across intervention groups; and (ii) The outcome measure was unlikely to be influenced by knowledge of the intervention received by study participants (i.e. is objective) or the outcome assessors were unaware of the intervention received by study

	participants; and (iii) Any error in measuring the outcome is unrelated to intervention status.
Moderate risk of bias (the study is sound for a non-randomized study with regard to this domain but cannot be considered comparable to a well-performed randomized trial)	(i) The methods of outcome assessment were comparable across intervention groups; and (ii) The outcome measure is only minimally influenced by knowledge of the intervention received by study participants; and (iii) Any error in measuring the outcome is only minimally related to intervention status.
Serious risk of bias (the study has some important problems)	(i) The methods of outcome assessment were not comparable across intervention groups; or (ii) The outcome measure was subjective (i.e. vulnerable to influence by knowledge of the intervention received by study participants); and The outcome was assessed by assessors aware of the intervention received by study participants; or (iii) Error in measuring the outcome was related to intervention status.
Critical risk of bias (the study is too problematic to provide any useful evidence on the effects of intervention)	The methods of outcome assessment were so different that they cannot reasonably be compared across intervention groups.
No information on which to base a judgement about risk of bias for this domain	No information is reported about the methods of outcome assessment.
**7. Reaching risk of bias judgements for bias in selection of the reported result**	
Low risk of bias (the study is comparable to a well-performed randomized trial with regard to this domain)	There is clear evidence (usually through examination of a pre-registered protocol or statistical analysis plan) that all reported results correspond to all intended outcomes, analyses and sub-cohorts.
Moderate risk of bias (the study is sound for a non-randomized study with regard to this domain but cannot be considered comparable to a well-performed randomized trial)	(i) The outcome measurements and analyses are consistent with an a priori plan; or are clearly defined and both internally and externally consistent; and (ii) There is no indication of selection of the reported analysis from among

	multiple analyses; and (iii) There is no indication of selection of the cohort or subgroups for analysis and reporting on the basis of the results.
Serious risk of bias (the study has some important problems)	(i) Outcomes are defined in different ways in the methods and results sections, or in different publications of the study; or (ii) There is a high risk of selective reporting from among multiple analyses; or (iii) The cohort or subgroup is selected from a larger study for analysis and appears to be reported on the basis of the results.
Critical risk of bias (the study is too problematic to provide any useful evidence on the effects of intervention)	(i) There is evidence or strong suspicion of selective reporting of results; and (ii) The unreported results are likely to be substantially different from the reported results.
No information on which to base a judgement about risk of bias for this domain.	There is too little information to make a judgement (for example if only an abstract is available for the study).

Table S4. Risk of bias of the studies included in this review through ROBINS-I assessment tool.

**Authors and Year of Publication**	**Signalling questions**	**Description**	**Response options**
Gahlert et al., 2012 [57]	**1. Bias due to confounding**		
	1.1 Is there potential for confounding of the effect of intervention in this study?<br>**If N/PN to 1.1:** the study can be considered to be at low risk of bias due to confounding and no further signalling questions need be considered	Occlusal and functional relations had been analyzed by clinical inspection.	Y / PY / PN / N
	**If Y/PY to 1.1**: determine whether there is a need to assess time-varying confounding:		
	1.2. Was the analysis based on splitting participants' follow up time according to intervention received?<br>**If N/PN**, answer questions relating to baseline confounding (1.4 to 1.6)<br>**If Y/PY**, go to question 1.3.		NA / Y / PY / PN / N / NI
	1.3. Were intervention discontinuations or switches likely to be related to factors that are prognostic for the outcome?<br>**If N/PN**, answer questions relating to baseline confounding (1.4 to 1.6)<br>**If Y/PY**, answer questions relating to both baseline and time-varying confounding (1.7 and 1.8)		NA / Y / PY / PN / N / NI
	Questions relating to baseline confounding only		
	1.4. Did the authors use an appropriate analysis method that controlled for all the important confounding domains?		NA / Y / PY / PN / N / NI
	1.5. **If Y/PY to 1.4**: Were confounding domains that were controlled for measured validly and reliably by the variables available in this study?		NA / Y / PY / PN / N / NI
	1.6. Did the authors control for any post-intervention variables that could have been affected by the intervention?		NA / Y / PY / PN / N / NI
	Questions relating to baseline and time-varying confounding		
	1.7. Did the authors use an appropriate analysis method that controlled for all the important confounding domains and for time-varying confounding?		NA / Y / PY / PN / N / NI
	1.8. **If Y/PY to 1.7**: Were confounding domains that were controlled for measured validly and reliably by the variables available in this study?		NA / Y / PY / PN / N / NI
	Risk of bias judgement		**Low** / Moderate / Serious / Critical / NI

	Optional: What is the predicted direction of bias due to confounding?		Favours experimental / Favours comparator / Unpredictable
	**2. Bias in selection of participants into the study**		
	2.1. Was selection of participants into the study (or into the analysis) based on participant characteristics observed after the start of intervention?<br>**If N/PN to 2.1:** go to 2.4	Selection of participants took place after start of intervention, as it was a retrospective study.	Y / PY / PN / N / NI
	2.2. **If Y/PY to 2.1**: Were the post-intervention variables that influenced selection likely to be associated with intervention?<br>2.3 **If Y/PY to 2.2**: Were the post-intervention variables that influenced selection likely to be influenced by the outcome or a cause of the outcome?		NA / Y / PY / PN / N / NI<br><br>NA / Y / PY / PN / N / NI
	2.4. Do start of follow-up and start of intervention coincide for most participants?		Y / PY / PN / N / NI
	2.5. **If Y/PY to 2.2 and 2.3, or N/PN to 2.4**: Were adjustment techniques used that are likely to correct for the presence of selection biases?		NA / Y / PY / PN / N / NI
	Risk of bias judgement		**Low** / Moderate / Serious / Critical / NI
	Optional: What is the predicted direction of bias due to selection of participants into the study?		**Favours experimental** / Favours comparator / Towards null /Away from null / Unpredictable
	**3. Bias in classification of interventions**		
	3.1 Were intervention groups clearly defined?	Fractured dental implants.	Y / PY / PN / N / NI
	3.2 Was the information used to define intervention groups recorded at the start of the intervention?		Y / PY / PN / N / NI

	3.3 Could classification of intervention status have been affected by knowledge of the outcome or risk of the outcome?	Analysis was limited to microscopic evaluation of fractured implants.	Y / PY / PN / N / NI
	Risk of bias judgement		**Low** / Moderate / Serious / Critical / NI
	Optional: What is the predicted direction of bias due to classification of interventions?		**Favours experimental** / Favours comparator / Towards null /Away from null / Unpredictable
	**4. Bias due to deviations from intended interventions**		
	If your aim for this study is to assess the effect of assignment to intervention, answer questions 4.1 and 4.2		
	4.1. Were there deviations from the intended intervention beyond what would be expected in usual practice?	All interventions were performed according to clinical practice.	Y / PY / PN / N / NI
	4.2. **If Y/PY to 4.1**: Were these deviations from intended intervention unbalanced between groups *and* likely to have affected the outcome?		NA / Y / PY / PN / N / NI
	If your aim for this study is to assess the effect of starting and adhering to intervention, answer questions 4.3 to 4.6		
	4.3. Were important co-interventions balanced across intervention groups?		Y / PY / PN / N / NI
	4.4. Was the intervention implemented successfully for most participants?		Y / PY / PN / N / NI
	4.5. Did study participants adhere to the assigned intervention regimen?		Y / PY / PN / N / NI
	4.6. **If N/PN to 4.3, 4.4 or 4.5**: Was an appropriate analysis used to estimate the effect of starting and adhering to the intervention?		NA / Y / PY / PN / N / NI
	Risk of bias judgement		**Low** / Moderate / Serious / Critical / NI

	Optional: What is the predicted direction of bias due to deviations from the intended interventions?		**<u>Favours experimental</u>** / Favours comparator / Towards null /Away from null / Unpredictable
	**5. Bias due to missing data**		
	5.1 Were outcome data available for all, or nearly all, participants?		<u>Y</u> / PY / PN / N / NI
	5.2 Were participants excluded due to missing data on intervention status?		Y / PY / PN / <u>N</u> / NI
	5.3 Were participants excluded due to missing data on other variables needed for the analysis?		Y / PY / PN / <u>N</u> / NI
	5.4 **If PN/N to 5.1, or Y/PY to 5.2 or 5.3**: Are the proportion of participants and reasons for missing data similar across interventions?		NA / Y / PY / PN / N / NI
	5.5 **If PN/N to 5.1, or Y/PY to 5.2 or 5.3**: Is there evidence that results were robust to the presence of missing data?		NA / Y / PY / PN / N / NI
	Risk of bias judgement		**<u>Low</u>** / Moderate / Serious / Critical / NI
	Optional: What is the predicted direction of bias due to missing data?		**<u>Favours experimental</u>** / Favours comparator / Towards null /Away from null / Unpredictable
	**6. Bias in measurement of outcomes**		
	6.1 Could the outcome measure have been influenced by knowledge of the intervention received?		Y / PY / PN / <u>N</u> / NI

	6.2 Were outcome assessors aware of the intervention received by study participants?	Retrospective study.	Y / PY / PN / N / NI
	6.3 Were the methods of outcome assessment comparable across intervention groups?		Y / PY / PN / N / NI
	6.4 Were any systematic errors in measurement of the outcome related to intervention received?		Y / PY / PN / N / NI
	Risk of bias judgement		**Low** / Moderate / Serious / Critical / NI
	Optional: What is the predicted direction of bias due to measurement of outcomes?		**Favours experimental** / Favours comparator / Towards null /Away from null / Unpredictable
	**7. Bias in selection of the reported result**		
	Is the reported effect estimate likely to be selected, on the basis of the results, from...		
	7.1. ... multiple outcome *measurements* within the outcome domain?		Y / PY / PN / N / NI
	7.2 ... multiple *analyses* of the intervention-outcome relationship?		Y / PY / PN / N / NI
	7.3 ... different *subgroups*?		Y / PY / PN / N / NI
	Risk of bias judgement		**Low** / Moderate / Serious / Critical / NI

	Optional: What is the predicted direction of bias due to selection of the reported result?		**Favours experimental** / Favours comparator / Towards null /Away from null / Unpredictable
	**Overall bias**		
	Risk of bias judgement		**Low** / Moderate / Serious / Critical / NI
	Optional: What is the overall predicted direction of bias for this outcome?		**Favours experimental** / Favours comparator / Towards null /Away from null / Unpredictable
Cha et al., 2013 [58]	**1. Bias due to confounding**		
	1.1 Is there potential for confounding of the effect of intervention in this study?<br>**If N/PN to 1.1:** the study can be considered to be at low risk of bias due to confounding and no further signalling questions need be considered	Occlusion was adjusted to provide similar occlusal contacts to the adjacent teeth in the intercuspal position with avoidance of lateral guiding contacts.	Y / PY / PN / N
	**If Y/PY to 1.1**: determine whether there is a need to assess time-varying confounding:		
	1.2. Was the analysis based on splitting participants' follow up time according to intervention received?<br>**If N/PN**, answer questions relating to baseline confounding (1.4 to 1.6)<br>**If Y/PY**, go to question 1.3.		NA / Y / PY / PN / N / NI

	1.3. Were intervention discontinuations or switches likely to be related to factors that are prognostic for the outcome?<br>**If N/PN**, answer questions relating to baseline confounding (1.4 to 1.6)<br>**If Y/PY**, answer questions relating to both baseline and time-varying confounding (1.7 and 1.8)		NA / Y / PY / PN / N / NI
	Questions relating to baseline confounding only		
	1.4. Did the authors use an appropriate analysis method that controlled for all the important confounding domains?		NA / Y / PY / PN / N / NI
	1.5. **If Y/PY to 1.4**: Were confounding domains that were controlled for measured validly and reliably by the variables available in this study?		NA / Y / PY / PN / N / NI
	1.6. Did the authors control for any post-intervention variables that could have been affected by the intervention?		NA / Y / PY / PN / N / NI
	Questions relating to baseline and time-varying confounding		
	1.7. Did the authors use an appropriate analysis method that controlled for all the important confounding domains and for time-varying confounding?		NA / Y / PY / PN / N / NI
	1.8. **If Y/PY to 1.7**: Were confounding domains that were controlled for measured validly and reliably by the variables available in this study?		NA / Y / PY / PN / N / NI
	Risk of bias judgement		**Low** / Moderate / Serious / Critical / NI
	Optional: What is the predicted direction of bias due to confounding?		**Favours experimental** / Favours comparator / Unpredictable
	**2. Bias in selection of participants into the study**		
	2.1. Was selection of participants into the study (or into the analysis) based on participant characteristics observed after the start of intervention?<br>**If N/PN to 2.1:** go to 2.4	Selection of participants took place after start of intervention, as it was a retrospective study.	Y / PY / PN / N / NI
	2.2. **If Y/PY to 2.1**: Were the post-intervention variables that influenced selection likely to be associated with intervention?<br>2.3 **If Y/PY to 2.2**: Were the post-intervention variables that influenced selection likely to be influenced by the outcome or a cause of the outcome?		NA / Y / PY / PN / N / NI<br><br>NA / Y / PY / PN / N / NI

	2.4. Do start of follow-up and start of intervention coincide for most participants?		Y / PY / PN / N / NI
	2.5. **If Y/PY to 2.2 and 2.3, or N/PN to 2.4**: Were adjustment techniques used that are likely to correct for the presence of selection biases?		NA / Y / PY / PN / N / NI
	Risk of bias judgement		**Low** / Moderate / Serious / Critical / NI
	Optional: What is the predicted direction of bias due to selection of participants into the study?		**Favours experimental** / Favours comparator / Towards null /Away from null / Unpredictable
	**3. Bias in classification of interventions**		
	3.1 Were intervention groups clearly defined?	Fractured dental implants.	Y / PY / PN / N / NI
	3.2 Was the information used to define intervention groups recorded at the start of the intervention?		Y / PY / PN / N / NI
	3.3 Could classification of intervention status have been affected by knowledge of the outcome or risk of the outcome?	The analysis was limited to the assessment of the long-term cumulative survival rate and the effect of clinical variables.	Y / PY / PN / N / NI
	Risk of bias judgement		**Low** / Moderate / Serious / Critical / NI

	Optional: What is the predicted direction of bias due to classification of interventions?		**Favours experimental** / Favours comparator / Towards null /Away from null / Unpredictable
	**4. Bias due to deviations from intended interventions**		
	If your aim for this study is to assess the effect of assignment to intervention, answer questions 4.1 and 4.2		
	4.1. Were there deviations from the intended intervention beyond what would be expected in usual practice?	All interventions were performed according to clinical practice.	Y / PY / PN / N / NI
	4.2. **If Y/PY to 4.1**: Were these deviations from intended intervention unbalanced between groups *and* likely to have affected the outcome?		NA / Y / PY / PN / N / NI
	If your aim for this study is to assess the effect of starting and adhering to intervention, answer questions 4.3 to 4.6		
	4.3. Were important co-interventions balanced across intervention groups?		Y / PY / PN / N / NI
	4.4. Was the intervention implemented successfully for most participants?		Y / PY / PN / N / NI
	4.5. Did study participants adhere to the assigned intervention regimen?		Y / PY / PN / N / NI
	4.6. **If N/PN to 4.3, 4.4 or 4.5**: Was an appropriate analysis used to estimate the effect of starting and adhering to the intervention?		NA / Y / PY / PN / N / NI
	Risk of bias judgement		**Low** / Moderate / Serious / Critical / NI

	Optional: What is the predicted direction of bias due to deviations from the intended interventions?		**Favours experimental** / Favours comparator / Towards null /Away from null / Unpredictable
	**5. Bias due to missing data**		
	5.1 Were outcome data available for all, or nearly all, participants?		Y / PY / PN / N / NI
	5.2 Were participants excluded due to missing data on intervention status?		Y / PY / PN / N / NI
	5.3 Were participants excluded due to missing data on other variables needed for the analysis?		Y / PY / PN / N / NI
	5.4 **If PN/N to 5.1, or Y/PY to 5.2 or 5.3**: Are the proportion of participants and reasons for missing data similar across interventions?		NA / Y / PY / PN / N / NI
	5.5 **If PN/N to 5.1, or Y/PY to 5.2 or 5.3**: Is there evidence that results were robust to the presence of missing data?		NA / Y / PY / PN / N / NI
	Risk of bias judgement		**Low** / Moderate / Serious / Critical / NI
	Optional: What is the predicted direction of bias due to missing data?		**Favours experimental** / Favours comparator / Towards null /Away from null / Unpredictable
	**6. Bias in measurement of outcomes**		
	6.1 Could the outcome measure have been influenced by knowledge of the intervention received?		Y / PY / PN / N / NI

	6.2 Were outcome assessors aware of the intervention received by study participants?	Retrospective study.	Y / PY / PN / N / NI
	6.3 Were the methods of outcome assessment comparable across intervention groups?		Y / PY / PN / N / NI
	6.4 Were any systematic errors in measurement of the outcome related to intervention received?		Y / PY / PN / N / NI
	Risk of bias judgement		**Low** / Moderate / Serious / Critical / NI
	Optional: What is the predicted direction of bias due to measurement of outcomes?		**Favours experimental** / Favours comparator / Towards null /Away from null / Unpredictable
	**7. Bias in selection of the reported result**		
	Is the reported effect estimate likely to be selected, on the basis of the results, from...		
	7.1. ... multiple outcome *measurements* within the outcome domain?		Y / PY / PN / N / NI
	7.2 ... multiple *analyses* of the intervention-outcome relationship?		Y / PY / PN / N / NI
	7.3 ... different *subgroups*?		Y / PY / PN / N / NI
	Risk of bias judgement		**Low** / Moderate / Serious / Critical / NI

	Optional: What is the predicted direction of bias due to selection of the reported result?		**Favours experimental** / Favours comparator / Towards null /Away from null / Unpredictable
	**Overall bias**		
	Risk of bias judgement		**Low** / Moderate / Serious / Critical / NI
	Optional: What is the overall predicted direction of bias for this outcome?		**Favours experimental** / Favours comparator / Towards null /Away from null / Unpredictable
Tabrizi et al., 2017 [59]	**1. Bias due to confounding**		
	1.1 Is there potential for confounding of the effect of intervention in this study?<br>**If N/PN to 1.1:** the study can be considered to be at low risk of bias due to confounding and no further signalling questions need be considered	Patients with fractured dental implants due to trauma were excluded.	Y / PY / PN / N
	**If Y/PY to 1.1**: determine whether there is a need to assess time-varying confounding:		
	1.2. Was the analysis based on splitting participants' follow up time according to intervention received?<br>**If N/PN**, answer questions relating to baseline confounding (1.4 to 1.6)<br>**If Y/PY**, go to question 1.3.		NA / Y / PY / PN / N / NI
	1.3. Were intervention discontinuations or switches likely to be related to factors that are prognostic for the outcome?<br>**If N/PN**, answer questions relating to baseline confounding (1.4 to 1.6)<br>**If Y/PY**, answer questions relating to both baseline and time-varying confounding (1.7 and 1.8)		NA / Y / PY / PN / N / NI

	Questions relating to baseline confounding only		
	1.4. Did the authors use an appropriate analysis method that controlled for all the important confounding domains?		NA / Y / PY / PN / N / NI
	1.5. **If Y/PY to 1.4**: Were confounding domains that were controlled for measured validly and reliably by the variables available in this study?		NA / Y / PY / PN / N / NI
	1.6. Did the authors control for any post-intervention variables that could have been affected by the intervention?		NA / Y / PY / PN / N / NI
	Questions relating to baseline and time-varying confounding		
	1.7. Did the authors use an appropriate analysis method that controlled for all the important confounding domains and for time-varying confounding?		NA / Y / PY / PN / N / NI
	1.8. **If Y/PY to 1.7**: Were confounding domains that were controlled for measured validly and reliably by the variables available in this study?		NA / Y / PY / PN / N / NI
	Risk of bias judgement		**Low** / Moderate / Serious / Critical / NI
	Optional: What is the predicted direction of bias due to confounding?		**Favours experimental** / Favours comparator / Unpredictable
	**2. Bias in selection of participants into the study**		
	2.1. Was selection of participants into the study (or into the analysis) based on participant characteristics observed after the start of intervention?<br>**If N/PN to 2.1:** go to 2.4	Selection of participants took place after start of intervention, as it was a retrospective study.	Y / PY / PN / N / NI
	2.2. **If Y/PY to 2.1**: Were the post-intervention variables that influenced selection likely to be associated with intervention?<br>2.3 **If Y/PY to 2.2**: Were the post-intervention variables that influenced selection likely to be influenced by the outcome or a cause of the outcome?		NA / Y / PY / PN / N / NI<br><br>NA / Y / PY / PN / N / NI
	2.4. Do start of follow-up and start of intervention coincide for most participants?		Y / PY / PN / N / NI
	2.5. **If Y/PY to 2.2 and 2.3, or N/PN to 2.4**: Were adjustment techniques used that are likely to correct for the presence of selection biases?		NA / Y / PY / PN / N / NI

	Risk of bias judgement		**Low** / Moderate / Serious / Critical / NI
	Optional: What is the predicted direction of bias due to selection of participants into the study?		**Favours experimental** / Favours comparator / Towards null /Away from null / Unpredictable
	**3. Bias in classification of interventions**		
	3.1 Were intervention groups clearly defined?	Fractured dental implants.	Y / PY / PN / N / NI
	3.2 Was the information used to define intervention groups recorded at the start of the intervention?		Y / PY / PN / N / NI
	3.3 Could classification of intervention status have been affected by knowledge of the outcome or risk of the outcome?	The analysis was limited to assessing whether clinical variables could influence implant fractures.	Y / PY / PN / N / NI
	Risk of bias judgement		**Low** / Moderate / Serious / Critical / NI
	Optional: What is the predicted direction of bias due to classification of interventions?		**Favours experimental** / Favours comparator / Towards null /Away from null / Unpredictable
	**4. Bias due to deviations from intended interventions**		
	If your aim for this study is to assess the effect of assignment to intervention, answer questions 4.1 and 4.2		

	4.1. Were there deviations from the intended intervention beyond what would be expected in usual practice?	All interventions were performed according to clinical practice.	Y / PY / PN / <u>N</u> / NI
	4.2. **If Y/PY to 4.1**: Were these deviations from intended intervention unbalanced between groups *and* likely to have affected the outcome?		NA / Y / PY / PN / N / NI
	If your aim for this study is to assess the effect of starting and adhering to intervention, answer questions 4.3 to 4.6		
	4.3. Were important co-interventions balanced across intervention groups?		Y / PY / PN / N / NI
	4.4. Was the intervention implemented successfully for most participants?		Y / PY / PN / N / NI
	4.5. Did study participants adhere to the assigned intervention regimen?		Y / PY / PN / N / NI
	4.6. **If N/PN to 4.3, 4.4 or 4.5**: Was an appropriate analysis used to estimate the effect of starting and adhering to the intervention?		NA / Y / PY / PN / N / NI
	Risk of bias judgement		**<u>Low</u>** / Moderate / Serious / Critical / NI
	Optional: What is the predicted direction of bias due to deviations from the intended interventions?		**<u>Favours experimental</u>** / Favours comparator / Towards null /Away from null / Unpredictable
	**5. Bias due to missing data**		
	5.1 Were outcome data available for all, or nearly all, participants?		<u>Y</u> / PY / PN / N / NI
	5.2 Were participants excluded due to missing data on intervention status?		Y / PY / PN / <u>N</u> / NI
	5.3 Were participants excluded due to missing data on other variables needed for the analysis?		Y / PY / PN / <u>N</u> / NI
	5.4 **If PN/N to 5.1, or Y/PY to 5.2 or 5.3**: Are the proportion of participants and reasons for missing data similar across interventions?		NA / Y / PY / PN / N / NI

	5.5 **If PN/N to 5.1, or Y/PY to 5.2 or 5.3**: Is there evidence that results were robust to the presence of missing data?		NA / Y / PY / PN / N / NI
	Risk of bias judgement		**Low** / Moderate / Serious / Critical / NI
	Optional: What is the predicted direction of bias due to missing data?		**Favours experimental** / Favours comparator / Towards null /Away from null / Unpredictable
	**6. Bias in measurement of outcomes**		
	6.1 Could the outcome measure have been influenced by knowledge of the intervention received?		Y / PY / PN / N / NI
	6.2 Were outcome assessors aware of the intervention received by study participants?	Retrospective study.	Y / PY / PN / N / NI
	6.3 Were the methods of outcome assessment comparable across intervention groups?		Y / PY / PN / N / NI
	6.4 Were any systematic errors in measurement of the outcome related to intervention received?		Y / PY / PN / N / NI
	Risk of bias judgement		**Low** / Moderate / Serious / Critical / NI
	Optional: What is the predicted direction of bias due to measurement of outcomes?		**Favours experimental** / Favours comparator / Towards null /Away from null / Unpredictable

	**7. Bias in selection of the reported result**		
	Is the reported effect estimate likely to be selected, on the basis of the results, from...		
	7.1. ... multiple outcome *measurements* within the outcome domain?		Y / PY / PN / **N** / NI
	7.2 ... multiple *analyses* of the intervention-outcome relationship?		Y / PY / PN / **N** / NI
	7.3 ... different *subgroups*?		Y / PY / PN / **N** / NI
	Risk of bias judgement		**Low** / Moderate / Serious / Critical / NI
	Optional: What is the predicted direction of bias due to selection of the reported result?		**Favours experimental** / Favours comparator / Towards null /Away from null / Unpredictable
	**Overall bias**		
	Risk of bias judgement		**Low** / Moderate / Serious / Critical / NI
	Optional: What is the overall predicted direction of bias for this outcome?		**Favours experimental** / Favours comparator / Towards null /Away from null / Unpredictable
Lee et al., 2018	**1. Bias due to confounding**		

[60]	1.1 Is there potential for confounding of the effect of intervention in this study?<br>**If** N/PN **to 1.1:** the study can be considered to be at low risk of bias due to confounding and no further signalling questions need be considered	Regular maintenance care was provided to prevent biological, mechanical, and technical complications.	Y / PY / PN / N
	**If** Y/PY **to 1.1**: determine whether there is a need to assess time-varying confounding:		
	1.2. Was the analysis based on splitting participants' follow up time according to intervention received?<br>**If N/PN**, answer questions relating to baseline confounding (1.4 to 1.6)<br>**If Y/PY**, go to question 1.3.<br>1.3. Were intervention discontinuations or switches likely to be related to factors that are prognostic for the outcome?<br>**If N/PN**, answer questions relating to baseline confounding (1.4 to 1.6)<br>**If Y/PY**, answer questions relating to both baseline and time-varying confounding (1.7 and 1.8)		NA / Y / PY / PN / N / NI<br><br><br><br>NA / Y / PY / PN / N / NI
	Questions relating to baseline confounding only		
	1.4. Did the authors use an appropriate analysis method that controlled for all the important confounding domains?		NA / Y / PY / PN / N / NI
	1.5. **If** Y/PY **to 1.4**: Were confounding domains that were controlled for measured validly and reliably by the variables available in this study?		NA / Y / PY / PN / N / NI
	1.6. Did the authors control for any post-intervention variables that could have been affected by the intervention?		NA / Y / PY / PN / N / NI
	Questions relating to baseline and time-varying confounding		
	1.7. Did the authors use an appropriate analysis method that controlled for all the important confounding domains and for time-varying confounding?		NA / Y / PY / PN / N / NI
	1.8. **If** Y/PY **to 1.7**: Were confounding domains that were controlled for measured validly and reliably by the variables available in this study?		NA / Y / PY / PN / N / NI
	Risk of bias judgement		**Low** / Moderate / Serious / Critical / NI
	Optional: What is the predicted direction of bias due to confounding?		**Favours experimental** / Favours comparator / Unpredictable

	**2. Bias in selection of participants into the study**		
	2.1. Was selection of participants into the study (or into the analysis) based on participant characteristics observed after the start of intervention?<br>**If** N/PN **to 2.1:** go to 2.4	Selection of participants took place after start of intervention, as it was a retrospective study.	Y / PY / PN / N / NI
	2.2. **If Y/PY to 2.1**: Were the post-intervention variables that influenced selection likely to be associated with intervention?<br>2.3 **If Y/PY to 2.2**: Were the post-intervention variables that influenced selection likely to be influenced by the outcome or a cause of the outcome?		NA / Y / PY / PN / N / NI<br><br>NA / Y / PY / PN / N / NI
	2.4. Do start of follow-up and start of intervention coincide for most participants?		Y / PY / PN / N / NI
	2.5. **If Y/PY to 2.2 and 2.3, or N/PN to 2.4**: Were adjustment techniques used that are likely to correct for the presence of selection biases?		NA / Y / PY / PN / N / NI
	Risk of bias judgement		**Low** / Moderate / Serious / Critical / NI
	Optional: What is the predicted direction of bias due to selection of participants into the study?		**Favours experimental** / Favours comparator / Towards null /Away from null / Unpredictable
	**3. Bias in classification of interventions**		
	3.1 Were intervention groups clearly defined?	Fractured dental implants.	Y / PY / PN / N / NI
	3.2 Was the information used to define intervention groups recorded at the start of the intervention?		Y / PY / PN / N / NI
	3.3 Could classification of intervention status have been affected by knowledge of the outcome or risk of the outcome?	The analysis was limited to assessing the pattern of implant fractures and clinical factors.	Y / PY / PN / N / NI

	Risk of bias judgement		**Low** / Moderate / Serious / Critical / NI
	Optional: What is the predicted direction of bias due to classification of interventions?		**Favours experimental** / Favours comparator / Towards null /Away from null / Unpredictable
	**4. Bias due to deviations from intended interventions**		
	If your aim for this study is to assess the effect of assignment to intervention, answer questions 4.1 and 4.2		
	4.1. Were there deviations from the intended intervention beyond what would be expected in usual practice?	All interventions were performed according to clinical practice.	Y / PY / PN / N / NI
	4.2. **If Y/PY to 4.1**: Were these deviations from intended intervention unbalanced between groups *and* likely to have affected the outcome?		NA / Y / PY / PN / N / NI
	If your aim for this study is to assess the effect of starting and adhering to intervention, answer questions 4.3 to 4.6		
	4.3. Were important co-interventions balanced across intervention groups?		Y / PY / PN / N / NI
	4.4. Was the intervention implemented successfully for most participants?		Y / PY / PN / N / NI
	4.5. Did study participants adhere to the assigned intervention regimen?		Y / PY / PN / N / NI
	4.6. **If N/PN to 4.3, 4.4 or 4.5**: Was an appropriate analysis used to estimate the effect of starting and adhering to the intervention?		NA / Y / PY / PN / N / NI
	Risk of bias judgement		**Low** / Moderate / Serious / Critical / NI

	Optional: What is the predicted direction of bias due to deviations from the intended interventions?		**Favours experimental** / Favours comparator / Towards null /Away from null / Unpredictable
	**5. Bias due to missing data**		
	5.1 Were outcome data available for all, or nearly all, participants?		Y / PY / PN / N / NI
	5.2 Were participants excluded due to missing data on intervention status?		Y / PY / PN / N / NI
	5.3 Were participants excluded due to missing data on other variables needed for the analysis?		Y / PY / PN / N / NI
	5.4 **If PN/N to 5.1, or Y/PY to 5.2 or 5.3**: Are the proportion of participants and reasons for missing data similar across interventions?		NA / Y / PY / PN / N / NI
	5.5 **If PN/N to 5.1, or Y/PY to 5.2 or 5.3**: Is there evidence that results were robust to the presence of missing data?		NA / Y / PY / PN / N / NI
	Risk of bias judgement		**Low** / Moderate / Serious / Critical / NI
	Optional: What is the predicted direction of bias due to missing data?		**Favours experimental** / Favours comparator / Towards null /Away from null / Unpredictable
	**6. Bias in measurement of outcomes**		
	6.1 Could the outcome measure have been influenced by knowledge of the intervention received?		Y / PY / PN / N / NI

	6.2 Were outcome assessors aware of the intervention received by study participants?	Retrospective study.	Y / PY / PN / N / NI
	6.3 Were the methods of outcome assessment comparable across intervention groups?		Y / PY / PN / N / NI
	6.4 Were any systematic errors in measurement of the outcome related to intervention received?		Y / PY / PN / N / NI
	Risk of bias judgement		**Low** / Moderate / Serious / Critical / NI
	Optional: What is the predicted direction of bias due to measurement of outcomes?		**Favours experimental** / Favours comparator / Towards null /Away from null / Unpredictable
	**7. Bias in selection of the reported result**		
	Is the reported effect estimate likely to be selected, on the basis of the results, from...		
	7.1. ... multiple outcome *measurements* within the outcome domain?		Y / PY / PN / N / NI
	7.2 ... multiple *analyses* of the intervention-outcome relationship?		Y / PY / PN / N / NI
	7.3 ... different *subgroups*?		Y / PY / PN / N / NI
	Risk of bias judgement		**Low** / Moderate / Serious / Critical / NI

	Optional: What is the predicted direction of bias due to selection of the reported result?		**Favours experimental** / Favours comparator / Towards null /Away from null / Unpredictable
	**Overall bias**		
	Risk of bias judgement		**Low** / Moderate / Serious / Critical / NI
	Optional: What is the overall predicted direction of bias for this outcome?		**Favours experimental** / Favours comparator / Towards null /Away from null / Unpredictable
Stoichkov et al., 2018 [61]	**1. Bias due to confounding**		
	1.1 Is there potential for confounding of the effect of intervention in this study?<br>**If N/PN to 1.1:** the study can be considered to be at low risk of bias due to confounding and no further signalling questions need be considered	All patients who experienced mechanical complications were examined for presence of parafunctional activity.	Y / PY / PN / N
	**If Y/PY to 1.1**: determine whether there is a need to assess time-varying confounding:		
	1.2. Was the analysis based on splitting participants' follow up time according to intervention received?<br>**If N/PN**, answer questions relating to baseline confounding (1.4 to 1.6)<br>**If Y/PY**, go to question 1.3.		NA / Y / PY / PN / N / NI

	1.3. Were intervention discontinuations or switches likely to be related to factors that are prognostic for the outcome?<br>**If N/PN**, answer questions relating to baseline confounding (1.4 to 1.6)<br>**If Y/PY**, answer questions relating to both baseline and time-varying confounding (1.7 and 1.8)		NA / Y / PY / PN / N / NI
	Questions relating to baseline confounding only		
	1.4. Did the authors use an appropriate analysis method that controlled for all the important confounding domains?		NA / Y / PY / PN / N / NI
	1.5. **If Y/PY to 1.4**: Were confounding domains that were controlled for measured validly and reliably by the variables available in this study?		NA / Y / PY / PN / N / NI
	1.6. Did the authors control for any post-intervention variables that could have been affected by the intervention?		NA / Y / PY / PN / N / NI
	Questions relating to baseline and time-varying confounding		
	1.7. Did the authors use an appropriate analysis method that controlled for all the important confounding domains and for time-varying confounding?		NA / Y / PY / PN / N / NI
	1.8. **If Y/PY to 1.7**: Were confounding domains that were controlled for measured validly and reliably by the variables available in this study?		NA / Y / PY / PN / N / NI
	Risk of bias judgement		**Low** / Moderate / Serious / Critical / NI
	Optional: What is the predicted direction of bias due to confounding?		**Favours experimental** / Favours comparator / Unpredictable
	**2. Bias in selection of participants into the study**		
	2.1. Was selection of participants into the study (or into the analysis) based on participant characteristics observed after the start of intervention?<br>**If N/PN to 2.1:** go to 2.4	Selection of participants took place after start of intervention, as it was a retrospective study.	Y / PY / PN / N / NI
	2.2. **If Y/PY to 2.1**: Were the post-intervention variables that influenced selection likely to be associated with intervention?<br>2.3 **If Y/PY to 2.2**: Were the post-intervention variables that influenced selection likely to be influenced by the outcome or a cause of the outcome?		NA / Y / PY / PN / N / NI<br><br>NA / Y / PY / PN / N / NI

	2.4. Do start of follow-up and start of intervention coincide for most participants?		Y / PY / PN / N / NI
	2.5. **If Y/PY to 2.2 and 2.3, or N/PN to 2.4**: Were adjustment techniques used that are likely to correct for the presence of selection biases?		NA / Y / PY / PN / N / NI
	Risk of bias judgement		**Low** / Moderate / Serious / Critical / NI
	Optional: What is the predicted direction of bias due to selection of participants into the study?		**Favours experimental** / Favours comparator / Towards null /Away from null / Unpredictable
	**3. Bias in classification of interventions**		
	3.1 Were intervention groups clearly defined?	Fractured dental implants.	Y / PY / PN / N / NI
	3.2 Was the information used to define intervention groups recorded at the start of the intervention?		Y / PY / PN / N / NI
	3.3 Could classification of intervention status have been affected by knowledge of the outcome or risk of the outcome?	The analysis was limited to analyzing possible factors leading implant fractures.	Y / PY / PN / N / NI
	Risk of bias judgement		**Low** / Moderate / Serious / Critical / NI

	Optional: What is the predicted direction of bias due to classification of interventions?		**Favours experimental** / Favours comparator / Towards null /Away from null / Unpredictable
	**4. Bias due to deviations from intended interventions**		
	If your aim for this study is to assess the effect of assignment to intervention, answer questions 4.1 and 4.2		
	4.1. Were there deviations from the intended intervention beyond what would be expected in usual practice?	All interventions were performed according to clinical practice.	Y / PY / PN / N / NI
	4.2. **If Y/PY to 4.1**: Were these deviations from intended intervention unbalanced between groups *and* likely to have affected the outcome?		NA / Y / PY / PN / N / NI
	If your aim for this study is to assess the effect of starting and adhering to intervention, answer questions 4.3 to 4.6		
	4.3. Were important co-interventions balanced across intervention groups?		Y / PY / PN / N / NI
	4.4. Was the intervention implemented successfully for most participants?		Y / PY / PN / N / NI
	4.5. Did study participants adhere to the assigned intervention regimen?		Y / PY / PN / N / NI
	4.6. **If N/PN to 4.3, 4.4 or 4.5**: Was an appropriate analysis used to estimate the effect of starting and adhering to the intervention?		NA / Y / PY / PN / N / NI
	Risk of bias judgement		**Low** / Moderate / Serious / Critical / NI

	Optional: What is the predicted direction of bias due to deviations from the intended interventions?		**Favours experimental** / Favours comparator / Towards null /Away from null / Unpredictable
	**5. Bias due to missing data**		
	5.1 Were outcome data available for all, or nearly all, participants?		Y / PY / PN / N / NI
	5.2 Were participants excluded due to missing data on intervention status?		Y / PY / PN / N / NI
	5.3 Were participants excluded due to missing data on other variables needed for the analysis?		Y / PY / PN / N / NI
	5.4 **If PN/N to 5.1, or Y/PY to 5.2 or 5.3**: Are the proportion of participants and reasons for missing data similar across interventions?		NA / Y / PY / PN / N / NI
	5.5 **If PN/N to 5.1, or Y/PY to 5.2 or 5.3**: Is there evidence that results were robust to the presence of missing data?		NA / Y / PY / PN / N / NI
	Risk of bias judgement		**Low** / Moderate / Serious / Critical / NI
	Optional: What is the predicted direction of bias due to missing data?		**Favours experimental** / Favours comparator / Towards null /Away from null / Unpredictable
	**6. Bias in measurement of outcomes**		
	6.1 Could the outcome measure have been influenced by knowledge of the intervention received?		Y / PY / PN / N / NI

	6.2 Were outcome assessors aware of the intervention received by study participants?	Retrospective study.	Y / PY / PN / N / NI
	6.3 Were the methods of outcome assessment comparable across intervention groups?		Y / PY / PN / N / NI
	6.4 Were any systematic errors in measurement of the outcome related to intervention received?		Y / PY / PN / N / NI
	Risk of bias judgement		**Low** / Moderate / Serious / Critical / NI
	Optional: What is the predicted direction of bias due to measurement of outcomes?		**Favours experimental** / Favours comparator / Towards null /Away from null / Unpredictable
	**7. Bias in selection of the reported result**		
	Is the reported effect estimate likely to be selected, on the basis of the results, from...		
	7.1. ... multiple outcome *measurements* within the outcome domain?		Y / PY / PN / N / NI
	7.2 ... multiple *analyses* of the intervention-outcome relationship?		Y / PY / PN / N / NI
	7.3 ... different *subgroups*?		Y / PY / PN / N / NI
	Risk of bias judgement		**Low** / Moderate / Serious / Critical / NI

	Optional: What is the predicted direction of bias due to selection of the reported result?		**Favours experimental** / Favours comparator / Towards null /Away from null / Unpredictable
	**Overall bias**		
	Risk of bias judgement		**Low** / Moderate / Serious / Critical / NI
	Optional: What is the overall predicted direction of bias for this outcome?		**Favours experimental** / Favours comparator / Towards null /Away from null / Unpredictable
Lee et al., 2019 [62]	**1. Bias due to confounding**		
	1.1 Is there potential for confounding of the effect of intervention in this study?<br>**If N/PN to 1.1:** the study can be considered to be at low risk of bias due to confounding and no further signalling questions need be considered	Exclusion of implants with inaccurate chart recordings, external or 1-piece connection systems, supported removable prostheses, failure of initial or early osseointegration.	Y / PY / PN / N
	**If Y/PY to 1.1**: determine whether there is a need to assess time-varying confounding:		
	1.2. Was the analysis based on splitting participants' follow up time according to intervention received?<br>**If N/PN**, answer questions relating to baseline confounding (1.4 to 1.6)<br>**If Y/PY**, go to question 1.3.		NA / Y / PY / PN / N / NI

	1.3. Were intervention discontinuations or switches likely to be related to factors that are prognostic for the outcome?<br>**If N/PN**, answer questions relating to baseline confounding (1.4 to 1.6)<br>**If Y/PY**, answer questions relating to both baseline and time-varying confounding (1.7 and 1.8)		NA / Y / PY / PN / N / NI
	Questions relating to baseline confounding only		
	1.4. Did the authors use an appropriate analysis method that controlled for all the important confounding domains?		NA / Y / PY / PN / N / NI
	1.5. **If Y/PY to 1.4**: Were confounding domains that were controlled for measured validly and reliably by the variables available in this study?		NA / Y / PY / PN / N / NI
	1.6. Did the authors control for any post-intervention variables that could have been affected by the intervention?		NA / Y / PY / PN / N / NI
	Questions relating to baseline and time-varying confounding		
	1.7. Did the authors use an appropriate analysis method that controlled for all the important confounding domains and for time-varying confounding?		NA / Y / PY / PN / N / NI
	1.8. **If Y/PY to 1.7**: Were confounding domains that were controlled for measured validly and reliably by the variables available in this study?		NA / Y / PY / PN / N / NI
	Risk of bias judgement		**Low** / Moderate / Serious / Critical / NI
	Optional: What is the predicted direction of bias due to confounding?		**Favours experimental** / Favours comparator / Unpredictable
	**2. Bias in selection of participants into the study**		
	2.1. Was selection of participants into the study (or into the analysis) based on participant characteristics observed after the start of intervention?<br>**If N/PN to 2.1:** go to 2.4	Selection of participants took place after start of intervention, as it was a retrospective study.	Y / PY / PN / N / NI
	2.2. **If Y/PY to 2.1**: Were the post-intervention variables that influenced selection likely to be associated with intervention?<br>2.3 **If Y/PY to 2.2**: Were the post-intervention variables that influenced selection likely to be influenced by the outcome or a cause of the outcome?		NA / Y / PY / PN / N / NI<br><br>NA / Y / PY / PN / N / NI

	2.4. Do start of follow-up and start of intervention coincide for most participants?		Y / PY / PN / N / NI
	2.5. **If Y/PY to 2.2 and 2.3, or N/PN to 2.4**: Were adjustment techniques used that are likely to correct for the presence of selection biases?		NA / Y / PY / PN / N / NI
	Risk of bias judgement		**Low** / Moderate / Serious / Critical / NI
	Optional: What is the predicted direction of bias due to selection of participants into the study?		**Favours experimental** / Favours comparator / Towards null /Away from null / Unpredictable
	**3. Bias in classification of interventions**		
	3.1 Were intervention groups clearly defined?	Fractured dental implants.	Y / PY / PN / N / NI
	3.2 Was the information used to define intervention groups recorded at the start of the intervention?		Y / PY / PN / N / NI
	3.3 Could classification of intervention status have been affected by knowledge of the outcome or risk of the outcome?	The analysis was limited to assessing the fracture rate and risk indicators of internal connection implants.	Y / PY / PN / N / NI
	Risk of bias judgement		**Low** / Moderate / Serious / Critical / NI

	Optional: What is the predicted direction of bias due to classification of interventions?		**Favours experimental** / Favours comparator / Towards null /Away from null / Unpredictable
	**4. Bias due to deviations from intended interventions**		
	If your aim for this study is to assess the effect of assignment to intervention, answer questions 4.1 and 4.2		
	4.1. Were there deviations from the intended intervention beyond what would be expected in usual practice?	All interventions were performed according to clinical practice.	Y / PY / PN / N / NI
	4.2. **If Y/PY to 4.1**: Were these deviations from intended intervention unbalanced between groups *and* likely to have affected the outcome?		NA / Y / PY / PN / N / NI
	If your aim for this study is to assess the effect of starting and adhering to intervention, answer questions 4.3 to 4.6		
	4.3. Were important co-interventions balanced across intervention groups?		Y / PY / PN / N / NI
	4.4. Was the intervention implemented successfully for most participants?		Y / PY / PN / N / NI
	4.5. Did study participants adhere to the assigned intervention regimen?		Y / PY / PN / N / NI
	4.6. **If N/PN to 4.3, 4.4 or 4.5**: Was an appropriate analysis used to estimate the effect of starting and adhering to the intervention?		NA / Y / PY / PN / N / NI
	Risk of bias judgement		Low / Moderate / Serious / Critical / NI

	Optional: What is the predicted direction of bias due to deviations from the intended interventions?		Favours experimental / Favours comparator / Towards null /Away from null / Unpredictable
	**5. Bias due to missing data**		
	5.1 Were outcome data available for all, or nearly all, participants?		Y / PY / PN / N / NI
	5.2 Were participants excluded due to missing data on intervention status?		Y / PY / PN / N / NI
	5.3 Were participants excluded due to missing data on other variables needed for the analysis?		Y / PY / PN / N / NI
	5.4 **If PN/N to 5.1, or Y/PY to 5.2 or 5.3**: Are the proportion of participants and reasons for missing data similar across interventions?		NA / Y / PY / PN / N / NI
	5.5 **If PN/N to 5.1, or Y/PY to 5.2 or 5.3**: Is there evidence that results were robust to the presence of missing data?		NA / Y / PY / PN / N / NI
	Risk of bias judgement		**Low** / Moderate / Serious / Critical / NI
	Optional: What is the predicted direction of bias due to missing data?		**Favours experimental** / Favours comparator / Towards null /Away from null / Unpredictable
	**6. Bias in measurement of outcomes**		
	6.1 Could the outcome measure have been influenced by knowledge of the intervention received?		Y / PY / PN / N / NI

	6.2 Were outcome assessors aware of the intervention received by study participants?	Retrospective study.	Y / PY / PN / N / NI
	6.3 Were the methods of outcome assessment comparable across intervention groups?		Y / PY / PN / N / NI
	6.4 Were any systematic errors in measurement of the outcome related to intervention received?		Y / PY / PN / N / NI
	Risk of bias judgement		**Low** / Moderate / Serious / Critical / NI
	Optional: What is the predicted direction of bias due to measurement of outcomes?		**Favours experimental** / Favours comparator / Towards null /Away from null / Unpredictable
	**7. Bias in selection of the reported result**		
	Is the reported effect estimate likely to be selected, on the basis of the results, from...		
	7.1. ... multiple outcome *measurements* within the outcome domain?		Y / PY / PN / N / NI
	7.2 ... multiple *analyses* of the intervention-outcome relationship?		Y / PY / PN / N / NI
	7.3 ... different *subgroups*?		Y / PY / PN / N / NI
	Risk of bias judgement		**Low** / Moderate / Serious / Critical / NI

	Optional: What is the predicted direction of bias due to selection of the reported result?		**<u>Favours experimental</u>** / Favours comparator / Towards null /Away from null / Unpredictable
	**Overall bias**		
	Risk of bias judgement		**<u>Low</u>** / Moderate / Serious / Critical / NI
	Optional: What is the overall predicted direction of bias for this outcome?		**<u>Favours experimental</u>** / Favours comparator / Towards null /Away from null / Unpredictable

Table S5. NHLBI Quality Assessment Tool for Observational Cohort Studies.

NHLBI Quality Assessment Tool for Observational Cohort Studies																
**First Author et al., Year**	**Q1**	**Q2**	**Q3**	**Q4**	**Q5**	**Q6**	**Q7**	**Q8**	**Q9**	**Q10**	**Q11**	**Q12**	**Q13**	**Q14**	**Total Score**	**Quality Rating**
Gahlert et al., 2012 [57]	Y	Y	Y	Y	N	Y	Y	Y	Y	N	Y	N	Y	Y	11/14 (78.57%)	Good
Cha et al., 2013 [58]	Y	Y	Y	Y	N	Y	Y	Y	Y	N	Y	N	Y	Y	11/14 (78.57%)	Good
Tabrizi et al., 2017 [59]	Y	Y	Y	Y	N	Y	Y	Y	Y	N	Y	N	Y	Y	11/14 (78.57%)	Good
Lee et al., 2018 [60]	Y	Y	Y	Y	N	Y	Y	Y	Y	N	Y	N	Y	Y	11/14 (78.57%)	Good
Stoichkov et al., 2018 [61]	Y	Y	Y	Y	N	Y	Y	Y	Y	N	Y	N	Y	Y	11/14 (78.57%)	Good
Lee et al., 2019 [62]	Y	Y	Y	Y	N	Y	Y	Y	Y	N	Y	N	Y	Y	11/14 (78.57%)	Good

Q1: Was the research question or objective in this paper clearly stated?, Q2: Was the study population clearly specified and defined?, Q3: Was the participation rate of eligible persons at least 50%?, Q4: Were all the subjects selected or recruited from the same or similar populations (including the same time period)? Were inclusion and exclusion criteria for being in the study prespecified and applied uniformly to all participants?, Q5: Was a sample size justification, power description, or variance and effect estimates provided?, Q6: For the analyses in this paper, were the exposure(s) of interest measured prior to the outcome(s) being measured?, Q7: Was the timeframe sufficient so that one could reasonably expect to see an association between exposure and outcome if it existed?, Q8: For exposures that can vary in amount or level, did the study examine different levels of the exposure as related to the outcome (e.g., categories of exposure, or exposure measured as continuous variable)?, Q9: Were the exposure measures (independent variables) clearly defined, valid, reliable, and implemented consistently across all study participants?, Q10: Was the exposure(s) assessed more than once over time?, Q11: Were the outcome measures (dependent variables) clearly defined, valid, reliable, and implemented consistently across all study participants?, Q12: Were the outcome assessors blinded to the exposure status of participants?, Q13: Was loss to follow-up after baseline 20% or less?, Q14: Were key potential confounding variables measured and adjusted statistically for their impact on the relationship between exposure(s) and outcome(s)?; Total Score: Number of yes; CD: cannot be determined; NA: not applicable; NR: not reported; N: no; Y: yes. Quality Rating: Poor <50%, Fair 50–75%, Good ≥75%.

References

14 Bufalá Pérez, M.; Zubizarreta-Macho, Á.; Borrajo Sánchez, J.; Hernández Rodríguez, J.; Alonso Pérez-Barquero, J.; Riad Deglow, E.; Hernández Montero, S. Removal capability, implant-abutment connection damage and thermal effect using ultrasonic and drilling techniques for the extraction of fractured abutment screws: an in vitro study. *BMC oral health*. **2022**, *221*, 603.

15 Gehrke, S.A.; Dedavid, B.A.; Prados-Frutos, J. C. Effects of different switched or not-switched implant and abutment platform designs and marginal bone loss on fracture strength: An in vitro study. *J.Prosthet. Dent.* **2022**, *128*, 55–62.

16 Khorshidparast, S.; Akhlaghi, P.; Rouhi, G.; Barikani, H. Measurement of bone damage caused by quasi-static compressive loading-unloading to explore dental implants stability: Simultaneous use of in-vitro tests, μ-CT images, and digital volume correlation. *J Mech Behav Biomed Mater*. **2023**, *138*, 105566.

17 Leitão-Almeida, B.; Camps-Font, O.; Correia, A.; Mir-Mari, J.; Figueiredo, R.; Valmaseda-Castellón, E. Effect of crown to implant ratio and implantoplasty on the fracture resistance of narrow dental implants with marginal bone loss: an in vitro study. *BMC oral health*. **2020**, *20*, 329.

18 Jorio, I.C.; Stawarczyk, B.; Attin, T.; Schmidlin, P.R.; Sahrmann, P. Reduced fracture load of dental implants after implantoplasty with different instrumentation sequences. An in vitro study. *Clin Oral Implants Res*. **2021**, *32*, 881–892.

19 Aramburú, J.S.; Gehrke, S.A.; Dedavid, B.A.; Treichel, T.L.E.; Pippi, N.L. Correlation of Fracture Resistance of Dental Implants and Bite Force in Dogs described in the literature: An In VitroStudy. *J Vet Dent*. **2021**, *38*, 75–80.

20 Bordin, D.; Bergamo, E.T.P.; Fardin, V.P.; Coelho, P.G.; Bonfante, E.A. Fracture strength and probability of survival of narrow and extra-narrow dental implants after fatigue testing: In vitro and in silico analysis. *J Mech Behav Biomed Mater*. **2021**, *71*, 244–249.

21 Kim, Y.J.; Ko, K.H.; Park, C.J.; Cho, L.R.; Huh, Y.H. Connector design effects on the in vitro fracture resistance of 3-unit monolithic prostheses produced from 4 CAD-CAM materials. *J Prosthet. Dent*. **2022**, *128*, 1319.e1–1319.e10.

22 Streckbein, P.; Wilbrand, J.F.; Kähling, C.; Pons-Kühnemann, J.; Rehmann, P.; Wöstmann, B.; Howaldt, H.P.; Möhlhenrich, S.C. Evaluation of the surface damage of dental implants caused by different surgical protocols: an in vitro study. *Int J Oral Maxillofac Surg*. **2019**, *48*, 971–981.

23 Burkhardt, F.; Spies, B.C.; Riemer, L.; Adolfsson, E.; Doerken, S.; Kohal, R.J. Fracture resistance and crystal phase transformation of a one- and a two-piece zirconia implant with and without simultaneous loading and aging-An in vitro study. *Clin Oral Implants Res*. **2021**, *32*, 1288–1298.

24 Gehrke, S.A.; Bonachela, W.C.; Lopes Moreno, J.M.; Orlato Rossetti, P.H.; Cortellari, G.C.; Dedavid, B.A.; Calvo-Guirado, J.L. Zirconium Oxide Three-Unit Fixed Partial Denture Frameworks Supported by Dental Implants in Acceptable and Reduced Interocclusal Space Possibilities: Pilot In Vitro Fracture Strength and Fractographic Analyses. *Int J Oral Maxillofac Implants*. **2019**, *34*, 337–342.

25 Agustín-Panadero, R.; Baixauli-López, M.; Gómez-Polo, M.; Cabanes-Gumbau, G.; Senent-Vicente, G.; Roig-Vanaclocha, A. In vitro comparison of the efficacy of two fractured implant-prosthesis screw extraction methods: Conventional versus mechanical. *J Prosthet Dent*. **2020**, *124*, 720–726.

26 Leitão-Almeida, B.; Camps-Font, O.; Correia, A.; Mir-Mari, J.; Figueiredo, R.; Valmaseda-Castellón, E. Effect of bone loss on the fracture resistance of narrow dental implants after implantoplasty. An in vitro study. *Med Oral Patol Oral Cir Bucal*. **2021**, *26*, e611–e618.

27 Bauer, R.; Zacher, J.; Strasser, T.; Rosentritt, M. In vitro performance and fracture resistance of interim conventional or CAD-CAM implant-supported screw- or cement-retained anterior fixed partial dentures. *J Prosthet Dent*. **2021**, *126*, 575–580.

28 Foong, J.K.; Judge, R.B.; Palamara, J.E.; Swain, M.V. Fracture resistance of titanium and zirconia abutments: an in vitro study. *J Prosthet Dent.* **2013**, *109*, 304–312.

29 El-Mahdy, M.; Aboelfadl, A.; Ahmed, F.; El-Banna, A.; Wahsh, M. Strain gauge analysis and fracture resistance of implant-supported PEKK hybrid abutments restored with two crown materials: An in vitro study. *Dent Med Probl*. **2023**, *60*, 497–503.
30 Kono, K.; Kurihara, D.; Suzuki, Y.; Ohkubo, C. In vitro assessment of mandibular single/two implant-retained overdentures using stress-breaking attachments. *Implant Dent*. **2014**, *23*, 456–462.
31 Asl, H.G.; Alsaran, A. In vitro comparison of commercial and ultrafine-grained titanium osteosynthesis miniplates used on mandibular fractures. *Dent Med Probl*. **2020**, *57*, 351–358.
32 Emam, M.; Arafa, A.M. Stress distribution and fracture resistance of green reprocessed polyetheretherketone (PEEK) single implant crown restorations compared to unreprocessed PEEK and Zirconia: an in-vitro study. *BMC oral health*, **2023**, *23*, 275.
33 Igarashi, K.; Afrashtehfar, K.I.; Schimmel, M.; Gazzaz, A.; Brägger, U. Performance of a repair service set for the retrieval of fractured abutment screws: a pilot in vitro study. *Int J Oral Maxillofac Implants*. **2019**, *34*, 567–573.
34 Freitas, D.Q.; Vasconcelos, T.V.; Noujeim, M. Diagnosis of vertical root fracture in teeth close and distant to implant: an in vitro study to assess the influence of artifacts produced in cone beam computed tomography. *Clin Oral Investig*. **2019**, *23*, 1263–1270.
35 Zhai, Z.; Nakano, T.; Chen, Y.; Watanabe, S.; Matsuoka, T.; Ishigaki, S. Implant deformation and implant-abutment fracture resistance after standardized artificial aging: An in vitro study. *Clin Implant Dent Relat Res*. **2023**, *25*, 107–117.
36 Vult von Steyern, P.; Kokubo, Y.; Nilner, K. Use of abutment-teeth vs. dental implants to support all-ceramic fixed partial dentures: an in-vitro study on fracture strength. *Swed Dent J*. **2005**, *29*, 53–60.
37 Gehrke, S.A.; Souza Dos Santos Vianna, M.; Dedavid, B.A. Influence of bone insertion level of the implant on the fracture strength of different connection designs: an in vitro study. *Clin Oral Investig*. **2014**, *18*, 715–720.
38 Chong, K.K.; Palamara, J.; Wong, R.H.; Judge, R.B. Fracture force of cantilevered zirconia frameworks: an in vitro study. *J Prosthet Dent*. **2014**, *112*, 849–856.
39 Kohal, R. J.; Wolkewitz, M.; Tsakona, A. The effects of cyclic loading and preparation on the fracture strength of zirconium-dioxide implants: an in vitro investigation. *Clin Oral Implants Res*. **2011**, *22*, 808–814.
40 Bein, L.; Rauch, A.; Schmidt, M.; Rosentritt, M. In vitro fatigue and fracture testing of temporary materials from different manufacturing processes in implant-supported anterior crowns. *Clin Oral Investig*. **2023**, *27*, 4215–4224.
41 Lin, J.; Cai, P.; Zhuo, Y.; Lin, L.; Zheng, Z. Effect of abutment design on fracture resistance of resin-matrix ceramic crowns for dental implant restoration: an in vitro study. *BMC oral health*. **2023**, *23*, 410.
42 Rues, S.; Kappel, S.; Ruckes, D.; Rammelsberg, P.; Zenthöfer, A. Resistance to Fracture in Fixed Dental Prostheses Over Cemented and Screw-Retained Implant-Supported Zirconia Cantilevers in the Anterior Region: An In Vitro Study. *Int J Oral Maxillofac Implants*. **2020**, *35*, 521–529.
43 Katsavochristou, A.; Sierraalta, M.; Saglik, B.; Koumoulis, D.; George, F.; Razzoog, M. Implant Angulation Effect on the Fracture Resistance of Monolithic Zirconia Custom Abutments: An In Vitro Study. *J Prosthodont*. **2020**, *29*, 394–400.
44 Vahnström, M.; Johansson, P.H.; Svanborg, P.; Stenport, V.F. Comparison of porcelain veneer fracture in implant-supported fixed full-arch prostheses with a framework of either titanium, cobalt-chromium, or zirconia: An in vitro study. *Clin Exp Dent*. **2022**, *8*, 544–551.
45 Sailer, I.; Sailer, T.; Stawarczyk, B.; Jung, R.E.; Hämmerle, C.H. In vitro study of the influence of the type of connection on the fracture load of zirconia abutments with internal and external implant-abutment connections. *Int J Oral Maxillofac Implants*. **2009**, *24*, 850–858.

46 Schmitter, M.; Rammelsberg, P.; Lenz, J.; Scheuber, S.; Schweizerhof, K.; Rues, S. Teeth restored using fiber-reinforced posts: in vitro fracture tests and finite element analysis. *Acta Biomater*. **2010**, *6*, 3747–3754.
47 Ghodsi, S.; Tanous, M.; Hajimahmoudi, M.; Mahgoli, H. Effect of aging on fracture resistance and torque loss of restorations supported by zirconia and polyetheretherketone abutments: An in vitro study. *J Prosthet Dent*. **2021**, *125*, 501.e1–501.e6.
48 Gehrke S.A. Importance of Crown Height Ratios in Dental Implants on the Fracture Strength of Different Connection Designs: An In Vitro Study. *Clin Implant Dent Relat Res.* **2015**, *17*, 790–797.
49 Giner, S.; Bartolomé, J.F.; Gomez-Cogolludo, P.; Castellote, C.; Pradíes, G. Fatigue fracture resistance of titanium and chairside CAD-CAM zirconia implant abutments supporting zirconia crowns: An in vitro comparative and finite element analysis study. *J Prosthet Dent*. **2021**, *125*, 503.e1–503.e9.
50 Wilmes, B.; Panayotidis, A.; Drescher, D. Fracture resistance of orthodontic mini-implants: a biomechanical in vitro study. *Eur J Orthod*. **2011**, *33*, 396–401.
51 Att, W.; Kurun, S.; Gerds, T.; Strub, J.R. Fracture resistance of single-tooth implant-supported all-ceramic restorations: an in vitro study. *J Prosthet Dent*. **2006**, *95*, 111–116.
52 Moorthy, A.; Aljudaibi, S.; Donnelly-Swift, E.; Polyzois, I.; Grufferty, B. An in vitro evaluation of 2 methods for retrieving fractured abutment screw fragments from the intaglio of 4 different implant systems. *J Prosthet Dent*. **2022**, S0022-3913(22)00466-8.
53 Coppedê, A.R.; Bersani, E.; de Mattos, M.daG.; Rodrigues, R.C.; Sartori, I.A.; Ribeiro, R.F. Fracture resistance of the implant-abutment connection in implants with internal hex and internal conical connections under oblique compressive loading: an in vitro study. *Int J Prosthodont*. **2009**, *22*, 283–286.
54 Patankar, A.; Kheur, M.; Kheur, S.; Lakha, T.; Burhanpurwala, M. Fracture Resistance of Implant Abutments Following Abutment Alterations by Milling the Margins: An In Vitro Study. *J Oral Implantol*. **2016**, *42*, 464–468.
55 Bhagat, J.A.; Naganathan, V.; Krishnan, L.; Raj, D.; Prakash, R. Development of a new V-shaped implant with locking plates and screws for mandibular fracture fixation: an in vitro study using finite element analysis. *Br J Oral Maxillofac Surg*. **2019**, *57*, 805–807.
56 Takeshita, K.; Toia, M.; Jinno, Y.; Sumi, T.; Takahashi, T.; Halldin, A.; Jimbo, R. Implant Vertical Fractures Provoked by Laboratory Procedures: A Finite Element Analysis Inspired from Clinical Cases. *Implant Dent.* **2016**, *25*, 361–366.
57 Gahlert, M.; Burtscher, D.; Grunert, I.; Kniha, H.; Steinhauser, E. Failure analysis of fractured dental zirconia implants. *Clin Oral Implants Res.* **2012**, *23*, 287-93.
58 Cha, H.S.; Kim, Y.S.; Jeon, J.H.; Lee, J.H. Cumulative survival rate and complication rates of single-tooth implant; focused on the coronal fracture of fixture in the internal connection implant. *J Oral Rehabil.* **2013**, *40*, 595-602.
59 Tabrizi, R.; Behnia, H.; Taherian, S.; Hesami, N. What Are the Incidence and Factors Associated With Implant Fracture? *J Oral Maxillofac Surg.* **2017**, *75*, 1866-1872.
60 Lee, J.H.; Kim, Y.T.; Jeong, S.N.; Kim, N.H.; Lee, D.W. Incidence and pattern of implant fractures: A long-term follow-up multicenter study. *Clin Implant Dent Relat Res.* **2018**, *20*, 463-469.
61 Stoichkov, B.; Kirov, D. Analysis of the causes of dental implant fracture: A retrospective clinical study. *Quintessence Int.* **2018**, *49*, 279-286.
62 Lee, D.W.; Kim, N.H.; Lee, Y.; Oh, Y.A.; Lee, J.H.; You, H.K. Implant fracture failure rate and potential associated risk indicators: An up to 12-year retrospective study of implants in 5,124 patients. *Clin Oral Implants Res.* **2019**, *30*, 206-217.
70 Sterne JAC, Higgins JPT, Elbers RG, Reeves BC and the development group for ROBINS-I. Risk Of Bias In Non-randomized Studies of Interventions (ROBINS-I): detailed guidance, updated 12 October 2016. Available from http://www.riskofbias.info [accessed {12 January 2024}]
